# Animal lifestyle affects acceptable mass limits for attached tags

**DOI:** 10.1098/rspb.2021.2005

**Published:** 2021-10-27

**Authors:** Rory P. Wilson, Kayleigh A. Rose, Richard Gunner, Mark D. Holton, Nikki J. Marks, Nigel C. Bennett, Stephen H. Bell, Joshua P. Twining, Jamie Hesketh, Carlos M. Duarte, Neil Bezodis, Milos Jezek, Michael Painter, Vaclav Silovsky, Margaret C. Crofoot, Roi Harel, John P. Y. Arnould, Blake M. Allan, Desley A. Whisson, Abdulaziz Alagaili, D. Michael Scantlebury

**Affiliations:** ^1^ Max Planck Institute of Animal Behavior, D-78315 Radolfzell, Germany; ^2^ College of Science, Swansea University, Fabian Way, Swansea SA1 8EN, UK; ^3^ School of Biological Sciences, Queen's University Belfast, Belfast BT9 5DL, UK; ^4^ Mammal Research Institute, Department of Zoology and Entomology, University of Pretoria, Pretoria 0002, South Africa; ^5^ Red Sea Research Centre, King Abdullah University of Science and Technology, Thuwal 23955, Saudi Arabia; ^6^ Applied Sports, Technology, Exercise and Medicine (A-STEM) Research Centre, College of Engineering, Swansea University, Bay Campus, Swansea SA1 8EN, UK; ^7^ Department of Game Management and Wildlife Biology, Faculty of Forestry and Wood Sciences, Czech University of Life Sciences, Prague 165 00, Czech Republic; ^8^ Department for the Ecology of Animal Societies, Max Planck Institute of Animal Behavior, Bücklestraβe 5, Konstanz D-78467, Germany; ^9^ Germany and Department of Biology, University of Konstanz, Konstanz 78457, Germany; ^10^ School of Life and Environmental Sciences, Deakin University, Melbourne Burwood Campus, 221 Burwood Highway, Burwood, VC 3125, Victoria, Australia; ^11^ KSU Mammals Research Chair, Zoology Department, King Saud University, Riyadh, Saudi Arabia

**Keywords:** collar design, detriment, ethics, guidelines, tag mass

## Abstract

Animal-attached devices have transformed our understanding of vertebrate ecology. To minimize any associated harm, researchers have long advocated that tag masses should not exceed 3% of carrier body mass. However, this ignores tag forces resulting from animal movement. Using data from collar-attached accelerometers on 10 diverse free-ranging terrestrial species from koalas to cheetahs, we detail a tag-based acceleration method to clarify acceptable tag mass limits. We quantify animal athleticism in terms of fractions of animal movement time devoted to different collar-recorded accelerations and convert those accelerations to forces (acceleration × tag mass) to allow derivation of any defined force limits for specified fractions of any animal's active time. Specifying that tags should exert forces that are less than 3% of the gravitational force exerted on the animal's body for 95% of the time led to corrected tag masses that should constitute between 1.6% and 2.98% of carrier mass, depending on athleticism. Strikingly, in four carnivore species encompassing two orders of magnitude in mass (*ca* 2–200 kg), forces exerted by ‘3%' tags were equivalent to 4–19% of carrier body mass during moving, with a maximum of 54% in a hunting cheetah. This fundamentally changes how acceptable tag mass limits should be determined by ethics bodies, irrespective of the force and time limits specified.

## 1. Introduction

The use of animal-attached devices is transforming our understanding of wild animal ecology and behaviour [1,2]. Indeed, tags containing multiple sensors and position-determining systems have been used across scales of time and space to measure everything from the extraordinary details of high performance hunts in cheetahs [3], to vast cross-taxon comparisons of animal behaviour and space-use over whole oceans (e.g. [1,4]). A critical proviso is, however, that such devices do affect survival or change the behaviour of their carriers, for both animal welfare issues as well as for scientific rigor [5]. Defining acceptable device loads for animals is critical because even diminishingly small tags can cause detriment. For example, Saraux *et al*. [6] showed that the addition of flipper rings to penguins can affect their populations, with adults having a survival rate 16% lower than untagged conspecifics and producing 39% fewer chicks, presumed to be because of the tags increasing the drag force in these fast-swimming birds. Performance is relevant in this case because drag-dependent energy expenditure to swim increases with the cube of the speed [7].

Although consideration of the physics of drag has been shown to be a powerful framework with which to understand tag detriment in aquatic animals (e.g. [8,9]), drag is negligible in terrestrial (though not aerial) systems even though tag detriment in terrestrial animals has been widely reported and is multi-facetted [10]. Reported issues range from minor behavioural changes [11] through skin-, subcutaneous- and muscle damage with ulceration [12,13] to reduced movement speed [14] and dramatically increased mortality [15]. As with drag, we advocate that a force-based framework is necessary to help understand such detriment. Indeed, force is implicit in ethics-based recommendations for acceptable tag loads because, for example, a central tenet is that animal tag mass should never exceed 3% or 5% of the animal-carrier body mass [16], this being based on early observations that tags weighing less than 5% of animal body masses apparently caused no change in behaviour [17]. Importantly though, there are now numerous studies that have reported highly variable impacts of animals carrying tags of masses less than the 3–5% limit [18–21] for reasons that are not always clear [20–22]. Implicit in this limit is that consequences, most particularly the physical forces experienced by animals owing to tags, are similarly limited. This cannot be true because Newton showed that mass, force and acceleration are linked via *F* = ma, so animal performance, specifically their acceleration, will affect the tag forces applied to the carriers. Tag forces on the animal carrier can therefore be assessed by measuring acceleration experienced by the tag as the animal moves. Specifically, reference to Newton's force/mass acceleration formulation shows that any time the tag acceleration exceeds 1 *g* (corresponding to Earth's gravity), the carrier animal will be subject to correspondingly higher tag-derived forces. We note here though, that this necessitates gathering on-animal data because simple consideration of acceleration from rigid-non-living bodies is inappropriate for living systems composed of multiple interacting segments [23].

Here, we examine the forces exerted by collar-mounted tags on moving animals. We investigate four species within the order Carnivora in detail; lions *Panthera leo*, European badgers *Meles meles*, pine martens *Martes martes* and a cheetah *Acinonyx jubatus* (with body masses roughly spanning 2–200 kg) equipped with accelerometers undertaking their normal activities in the wild for 1–21 days. In particular, because gait is known to affect acceleration in body-mounted tags [24] we examined how walking, trotting and bounding affected the forces imposed on the animals by the tags. We also equipped six other species of mammal from diverse animal families (a cercopithecid, a phascolarctid, a phalagerid, a bovid, a cervid and a suid) with different lifestyles with accelerometers *in situ* for periods between 7 and 168 days to examine the general patterns of forces they exhibited and compared them to the carnivores.

Because the act of travelling is known to produce particularly high forces [25], we also carried out controlled trials with 12 domestic dogs *Canis familiaris* (2–45 kg) equipped with the same tags moving at defined speeds to investigate how movement speed, body mass and tag mass interact to affect tag forces.

We document how the forces imposed by the collars changed with activity across all these species and conditions. Based on this, we propose a method based on acceleration data that allows researchers to define the breadth of forces exerted by tags on animals and their relative frequency of occurrence. We show how this information can then be used to derive appropriately force-based acceptable limits for tag masses, recognizing the effect of animal lifestyle and athleticism.

## 2. Material and methods

### (a) Tag deployments on free-ranging species

We selected four species of free-living carnivores for detailed analysis, exemplifying about two orders of magnitude of mass; 10 lions *Panthera leo* (mean mass *ca* 152 kg), one cheetah *Acinonyx jubatus* (mass *ca* 41 kg), 10 badgers *Meles meles* (mean mass *ca* 9.1 kg) and five pine martens *Martes martes* (mean mass 1.9 kg), and fitted them with collar-mounted tri-axial accelerometers (‘Daily Diaries—Wildbyte Technologies (http://www.wildbytetechnologies.com/); measurement range 0–16 *g* (resolution 0.49 m*g*), recording frequency 40 Hz), all of which constituted less than 3% of the mass of the animal carriers (electronic supplementary material, table S1). Owing to the weighting of the loggers, and more particularly their associated batteries, the units and sensors were positioned on the underside of the collar although during movement the collars could rotate, which could occasionally, temporarily bring the measuring system off the ventral position. After being equipped, the animals roamed freely, behaving normally, for periods ranging between 3 and 21 days before the devices were recovered.

In addition to these, we also deployed collar-mounted accelerometers constituting less than 3% of the carrier mass (electronic supplementary material, table S1) on six select free-ranging animal species. We chose these species by capitalizing on available data from animals equipped with high temporal resolution acceleration tags on collars from different mammal families with varying lifestyles for comparison with the carnivores. The species and lifestyles were: a savannah-dwelling monkey—the olive baboon *Papio Anubis* (mean mass 15 kg, *n* = 5); an arboreal herbivorous marsupial—the koala *Phascolarctos cinereus* (mean mass 10.3 kg, *n* = 5); a nocturnal, semi-arboreal, herbivorous marsupial—the mountain brushtail possum *Trichosurus cunninghami* (mean mass 3.2 kg, *n* = 5); a grass-eating, desert-dwelling bovid—the Arabian oryx *Oryx leucoryx* (mean mass 74 kg, *n* = 5); a grass-eating, wood- and moor-dwelling cervid—the red deer *Cervus elaphus* (mean mass 135 kg, *n* = 5); and a forest-dwelling, omnivorous pig—the wild boar *Sus scrofa* (mean mass 67 kg, *n* = 5)*.* Extensive details on species-specific tagging procedures are included in the electronic supplementary material.

### (b) Trials with domestic dogs

Twelve domestic dogs (*Canis lupus domesticus*) of seven different breed combinations and three main body types (small, racers and northern breeds), ranging 2–45 kg in body mass (electronic supplementary material, table S2), were volunteered by their owners and the Royal Society for the Prevention of Cruelty to Animals (RSPCA) Llys Nini Wildlife Centre (Penllergaer, Wales) to take part in this study. Dog body masses were obtained from the most recent measurements taken by a veterinarian, or the RSPCA, and we measured body length, forelimb length and hindlimb length to the nearest cm. Two leather dog collars (short and long) of the same width were used to cover the range in dog neck size. Combinations of pre-prepared lead plates (up to 10 cm in length) and varying in mass (25, 35, 45, 50, 100, 150 and 175 g) were fashioned into collar loads equivalent to 1, 2 and 3% of each carrier dog's body mass. The lead plates were stacked, the longest of them (for the greatest masses) being bent to replicate a 10 cm section of the collar circumference and attached securely to the ventral collar along their full-length using Tesa® tape. A tri-axial accelerometer and its supporting battery (3.2 V lithium ion) were taped securely to the load. The tag and battery combined weighed 11.9 g and, in the absence of any additional load, were considered negligible in mass and used as a control (0% carrier body mass). All trials were approved by the Swansea University Animal Welfare Ethical Review Body (ethical approval number IP-1617-21D).

Each dog was encouraged to traverse along a 25 m stretch of level, short-cut grass at slow (walk/amble), moderate (pace/trot) and fast (canter/gallop) speeds (because gait affects acceleration signatures substantially [24]) wearing collar-tags equivalent to 0, 1, 2 and 3% of their body mass (12 gait and tag mass combinations) and trial order was randomized. Posts were spaced every 5 m along the track. A stopwatch was used to record the time taken (to the nearest s) for a dog to travel 20 m in order to calculate an average speed of travel (m s^−1^).

### (c) Data processing

In all cases of animals equipped with accelerometers, the three channels of raw acceleration data were converted to a single metric by calculating the vectorial sum of the acceleration following $\mathrm{Vect}\;\mathrm{sum}\;=\sqrt{\left(a_{x}^{2}+a_{y}^{2}+a_{z}^{2}\right)}$, where *a* is the instantaneous acceleration and the subscripts denote the different (orthogonally placed) acceleration axes. We chose to use the Vect sum rather than dynamic body acceleration (DBA) metrics [26] because DBA values do not represent peak accelerations owing to the gravity-based component being removed [27]. The specifics of the surge, heave and sway accelerations were not considered separately owing to some collar roll. In the case of the free-living carnivores, we examined how travel gait affected the Vect sum by plotting the cumulative frequency distribution from each species during periods of walking, trotting and bounding.

For the domestic dogs, we selected the maximum four peak accelerations in the Vect sum from the gait waveforms using the peak analysis tool in OriginLab (2020) to examine them as a function of average speed, gait, body mass and tag mass as a percentage of carrier body mass. We standardized the use of four peaks because at the highest speeds some dogs only had four full waveforms during the test stretch. The relative forces (% body mass) exerted by the tags on their animal carriers were calculated using *F* = ma, where *m* is the mass of the tag plus collar as a percentage of carrier mass and *a* is the acceleration (*g*).

### (d) Tag-based acceleration method

Finally, in a full cross-species comparison of the free-living animals, we plotted the cumulative frequency distribution of the Vect sum from each species during periods when they were active (by excluding periods where the acceleration signals were constant) to define the vector sum of the acceleration at species-specific 95% and 99% limits.

### (e) Statistical analyses

Linear mixed-effects models were conducted in R (v. 4.0.3, [28]) within the ‘lme4' package (v. 1.1-26) in order to investigate how the period between acceleration peaks, gait and body mass influenced peak accelerations across four species of wild carnivores, and separately in domestic dogs. Additionally, we investigated how travel speed (covariate), body mass (covariate), collar mass as a percentage of carrier body mass (fixed factor with four levels) and gait (fixed factor with three levels for slow, moderate and fast gaits) influenced peak accelerations and consequent forces exerted by the tags. Dog identity was included as a random factor in all models to account for repeated measures. All potential interaction effects were first investigated and a step-wise back-deletion of non-significant interaction terms was conducted. Standard model diagnostics were conducted in order to ensure that model assumptions were met (examining quantile-quantile plots and plotting the residuals against fitted values) and data transformations were conducted in order to meet assumptions where appropriate. The *F* statistic and marginal and conditional R^2^ were determined using the ‘car (3.0-5)' and ‘MuMIn (1.46.6)' packages, respectively. Coefficients for best-fit lines in the figures were extracted from the final outputs of the models.

## 3. Results

### (a) Changing acceleration according to activity in carnivores

**Figure 1.**
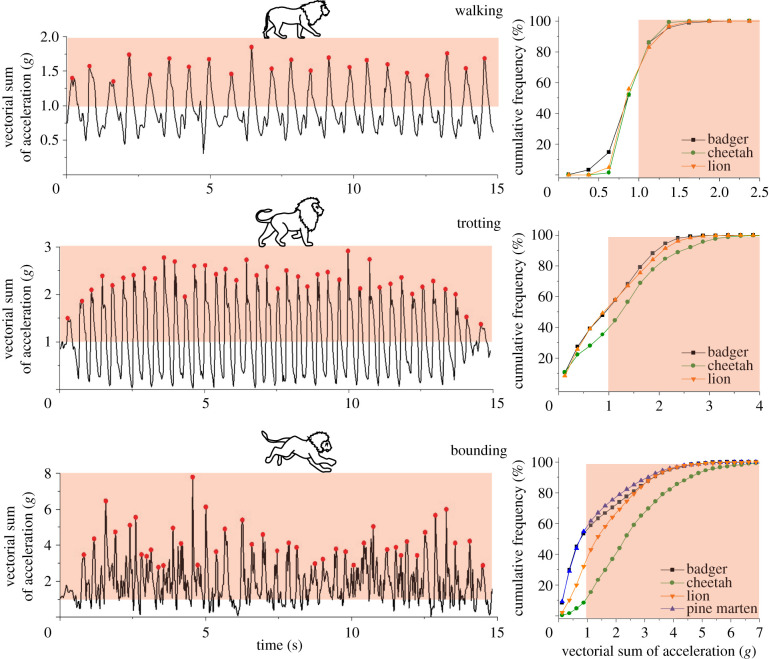
Acceleration signatures vary according to gait and lifestyle. Left-hand panels: acceleration signatures recorded by collar-mounted tags on a lion according to activity. The red areas show when the acceleration exceeded that of gravity (note the changing scales with gait). Right-hand panels: cumulative frequency of all acceleration values for four free-living carnivores according to gait. Note that the pine martens never walked or trotted. (Online version in colour.)

**Figure 2.**
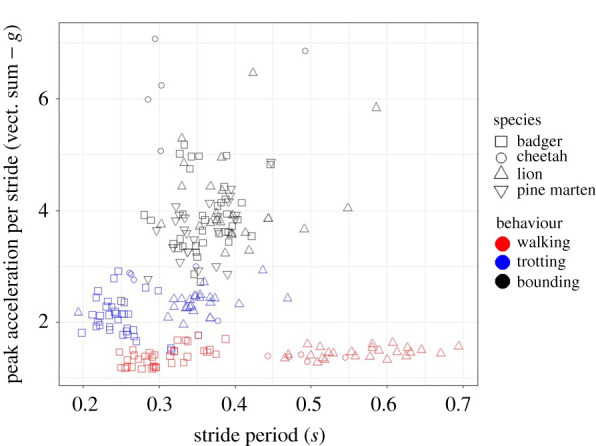
Body mass and stride period do not dictate peak tag acceleration. Distributions of peak amplitudes of (the vectorial sum of) accelerations and stride periods for four free-living carnivores (see symbols, with mean masses of *ca.* 2 kg, 9 kg, 41 kg and 152 kg for the pine martens, badgers, cheetah and lions, respectively) travelling using different assumed gaits (colours). Each individual point shows a mean from a duration of activity greater than 5 s from a single individual. See also the electronic supplementary material, figure S1 for similar data from domestic dogs. (Online version in colour.)

Accelerometer data for periods when our carnivores travelled, displayed clear peaks in the waveforms with measurable frequency and, summarized as a frequency distribution of the vectorial sum of the three orthogonal axes, showed tri-modal distributions except for the pine martens which were mono-modal. Following [29] and examination of videos of the study animals engaged in travelling using different gaits with measurable step frequency, we could ascertain that these corresponded to walking, trotting and bounding (e.g. figure 1, which also tallied with our direct and filmed observations of the domestic dogs below); these were further exemplified by variation in the amplitude in this acceleration metric (figure 2). Cumulative frequencies of all acceleration values showed increasing acceleration from walking through trotting to bounding and typically had a roughly logarithmic-type curve for all gaits and animals (figure 1). The percentage time during which the tags carried by the carnivores had acceleration exceeding 1 *g* during specified activity, varied between a mean minimum of 31% for walking badgers to 88% for bounding cheetahs (electronic supplementary material, table S3). Furthermore, while differences in species acceleration distributions were not readily apparent for their walking gaits, the percentage time during which acceleration was in excess of 1 *g* was greatest during bounding, with cheetahs showing the highest values in this category (green line with circles in figure 1). Mean peak accelerations per stride across species varied between 1.37 *g* (s.d. 0.05) and 6.25 *g* (s.d. 0.79) for walking and bounding cheetahs, respectively (electronic supplementary material, table S4). The maximum recorded value was 18.1 *g* in a cheetah assumed to be chasing prey.

Across the four species, gait was the main factor dictating peak acceleration (figure 2) and there were no significant effects of body mass, nor period between peaks (linear mixed-effects model: log period: *F*_1,210_ = 0.01, *p* = 0.908; gait: *F*_2,208_ = 1083.07, *p <* 0.0001; body mass: *F*_1,19_ = 3.00; *p* = 0.100; electronic supplementary material, table S5). The period between acceleration peaks was greater for larger species during slower gaits, but not for bounding (a linear mixed-effects model demonstrated a significant interaction effect between body mass and gait: *F*_2,209_ = 3.00, *p <* 0.0001; electronic supplementary material, table S5).

**Figure 3.**
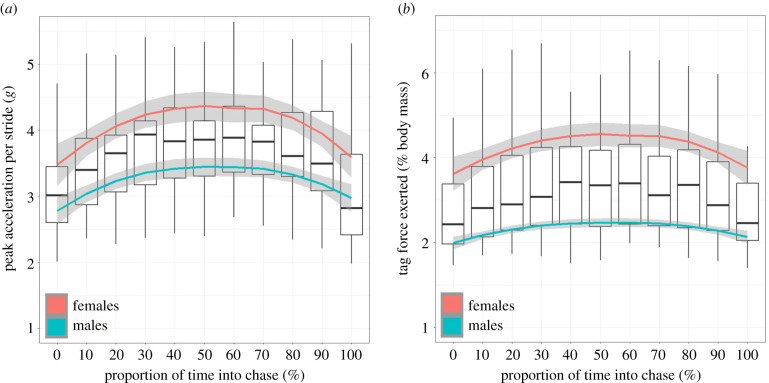
Hunting lions experience maximum tag forces mid-chase and show substantial inter-sex differences. Box and whisker plots (bold horizontal bars show means, boxes inter-quartile ranges (IQR) and whiskers 1.5 × IQR) of the: (*a*) vectorial sum of the acceleration peaks per bound (cf. figure 1), and (*b*) the tag-based forces exerted as a percentage of the gravitational force exerted on the animal's body (because our tag constituted 1.02% and 0.72% of the female and male body weights, respectively, see the electronic supplementary material, table S1) for lions chasing prey as a function of the percentage progression into the chase (considered to have started when bounding began). Red (upper) and blue (lower) lines show grand means for five females and five males, respectively. The maximum acceleration was 15.1 *g*, which would equate to a 3% tag exerting a force equivalent to 45.3% of the gravitational force exerted on the animal's body. (Online version in colour.)

There was also appreciable variation in the vectorial sum of the acceleration within gaits and between sexes, as exemplified by prey chases by lions. Here, mean peak acceleration per stride across females and males increased from about 3 *g* at the outset to a maximum of about 3.8 *g* before decreasing again (figure 3). However, female peak acceleration was approximately 1 *g* higher than males for the duration of the chase (figure 3). Given that females and males were wearing tags that amounted to 0.72% and 1.02% of their mean body masses, respectively (electronic supplementary material, table S1), this translates to tag-dependent forces corresponding to greater than 2% and greater than 4% of the gravitational force exerted on the animal's body masses, respectively (figure 3). In the case of the cheetah, which showed the highest peak vectorial acceleration sum of our study animals, a 3% tag would impose forces equivalent to 54% of the gravitational force exerted on the animal's body at this time.

In dogs, stride peak accelerations increased linearly with travel speed (electronic supplementary material, figure S2), but at greater rates with increasing relative tag mass (there was a significant interaction effect between travel speed and tag per cent body mass: *F*_3,500.77_ = 4.34_,_ *p* = 0.004; electronic supplementary material, table S6). There was also a significant interaction effect between gait and tag per cent body mass on stride peak accelerations (*F*_6,498.57_ = 4.34, *p* = 0.0002; electronic supplementary material, table S6). Peak tag accelerations ranged from 4 to 18 *g* during fast category (canter/gallop) trials in dogs wearing collar tags equivalent to 3% of their body mass (electronic supplementary material, figure S3). In this scenario, movement of the tag relative to the body (flapping/swinging) was exacerbated and, as a consequence, the force exerted by the tags ranged from 20–50% of the gravitational force exerted on the carrier animal's body mass (electronic supplementary material, figure S4).

Stride peak accelerations were largely invariant with body mass (*F*_1,10.12_ = 3.51, *p* = 0.090; electronic supplementary material, table S6) across dog breeds for any given gait (electronic supplementary material, figure S3). Consequently, the peak forces exerted by the tags were directly proportional to tag mass and body mass. Accordingly, relative tag forces (percentage of the gravitational force exerted on the carrier animal's body mass) were independent of carrier body mass (electronic supplementary material, table S6 and figure S4).

### (b) Using accelerometry to derive an over-arching tag-force rule

**Figure 4.**
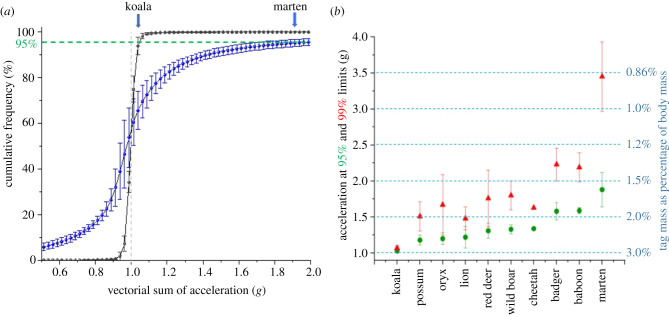
Defining tag mass limits based on cumulative time spent experiencing tag forces. Panel (*a*) shows the mean cumulative frequency (bars = s.d.) of the vectorial sum of the acceleration for two arboreal animals with very different athleticisms: five koalas (black line concentrated around 1 *g*) and five pine martens (blue line with much greater spread around 1 *g*). The 95% limit is shown by the dashed (green) line. Panel (*b*) shows these two species points (and the 99% limits in red triangles) adjacent to a broader species list highlighting variation in lifestyles. Assuming that a tag should only exert a force amounting to 3% of the gravitational force exerted on the carrier animal's body, the translation of these species-specific acceleration limits can be used to correct tag masses to be an appropriate percentage of the carrier animal mass (blue axis on the right). (Online version in colour.)

Although travelling is a major component across species, animal activity across all behaviours contributes to the acceleration, and therefore the tag force profiles, that animals experience. We produced cumulative frequency curves of the vectorial sum of the acceleration (cf. figure 1) for all 10 study species for periods when animals were considered active and these all showed a characteristic sigmoid pattern (figure 4*a*). These relationships were displaced further to the right as higher acceleration activities accounted for an increasing proportion of any animal's time (figure 4*a*). In order to have a scientifically robust acceptable threshold to limit the forces produced by a tag on an animal carrier, we suggest a tag-based acceleration method; that researchers should derive a similar cumulative acceleration profile for their study species and use a minimum of the 95% limits on the plot (although higher limits may be more appropriate). Assuming, in the case of our study animals, that these limits were intended to cater for a tag that should exert forces that are less than 3% of the gravitational force exerted on the animal's body, this limit would lead to corrected tag masses constituting between 1.6% and 2.98% of our study animals' masses (figure 4*b*). We note, however, that even these corrected tag masses would effectively exceed the 3% rule conditions for one-twentieth of the animals' active periods: the difference between the 95% and 99% thresholds for our study species indicates the extent of the force development for this period with some, such as the koalas, showing virtually no difference, whereas badgers, baboons and martens exhibited substantial differences (figure 4*b*). Importantly though, this method would allow researchers to define any tag force thresholds, not just 3%, and the times these were exceeded by the animal, not just 95%.

## 4. Discussion

The subject of detriment caused by tags on animals is complex because the general term ‘detriment’ has many facets [20], not least because the tag itself may cause the animal to move in an atypical manner, which may change how a device would affect an animal that did not respond to the device. One direct aspect that exemplifies this is, for example, measurable physical harm to the animal, such as pressure sores [12], the severity of which might be expected to depend on movement patterns. However, physical harm can also effectively occur if tagged animals or their offspring cannot balance energy budgets owing to compromised foraging stemming from tag interference [6,30,31]. Often, this is simply a result of higher movement costs or reduced performance in tagged animals as they travel [18]. This also means though, that precise limb kinematics may be different in travelling tagged animals, and this will affect acceleration signals recorded by animal-attached tags, which is relevant to a study such as ours. So, measurement affects performance [32] and we must bear this caveat in mind when we advocate that our tag data represent the norm of untagged animals. Against this, however, we can and should use proper physical frameworks to assess tag detriment because this is precisely what our tagged animals experience, whether their movement is ‘normal' or not, because we have specifically equipped them with the source of detriment. Indeed, this is the fundamental premise behind our work although the issue of what untagged animals may experience remains problematic [5].

A rigid vehicle accelerating in a straight-line only experiences acceleration in the longitudinal axis. By contrast, the multiple limb-propelled motion of an animal with a flexible body produces complex three-dimensional trunk accelerations owing to the changing limb accelerations [23] caused by multiple muscle groups that ultimately transfer mechanical energy and affect shock absorption [33], and the mechanical work conducted within each stride [34]. Ultimately, the magnitude of trunk accelerations depends on the combined acceleration of the limbs, and the masses of those limbs (cf. [23]). Thus, animals engaging in high performance activities are expected to produce high body accelerations, and have physiological and anatomical adaptations to enhance performance, such as fast twitch muscles [35], and tendons designed for greater storage and release [36], which will increase this. Through all these complexities, tags mounted on the trunk of an animal result in greater forces being imposed that scale linearly with the acceleration of the tag and its mass. Consideration of animal lifestyle then, can already inform prospective tag users of the likely scale-up of the tag forces beyond the 1 *g* normally considered for tag detriment because force = mass × acceleration, the repercussions of which are discussed below in terms of potential detriment. Consequently, the 3–5% mass limits for slow-moving animals, such as sloths (Bradipodidae) or koalas (Phascolarctidae) (figure 4), seem most appropriate, though this does not mean that tags will not affect the animals. Against this, the 3–5% mass limits may be less appropriate for pursuit predators, such as wild dogs (*Lycaon pictus*), regularly jumping animals like kangaroos (Macropodidae) or martens (Mustelidae) (figure 4) and rutting ungulates (Ungulata). Beyond that, in our small sample of carnivores at least, which nonetheless covers about two orders of magnitude in mass, it seems that peak acceleration associated with gait varies little with mass, although larger animals have longer stride periods (figure 2, cf. [37]). If these animals were to carry tags constituting 3% of their normal body mass, mean peak forces imposed by the tags would constitute *ca* 4.5%, 6% and 12% of the gravitational forces exerted on the animals' bodies for walking, trotting and bounding gaits at frequencies of between 1.6 and 4 times per second (for walking lions and trotting badgers, respectively; figure 2; electronic supplementary material, table S4). We also note how minor differences in sex-dependent tag masses coupled with differences in performance affect the forces imposed by the tags, as exemplified by the lions (figure 3) and how, were the tags in this study to constitute 3% of the animals' masses, the tag-based forces would scale up accordingly. Against all this, we recognize two important trends: (i) that as animals get larger, deployed tags on them are likely to be a smaller fraction of their mass anyway; but that (ii) despite miniaturization advances in tag technology, researchers continue to deploy systems that are around the 3–5% mass limit on smaller animals [21].

Importantly, tag attachment is relevant in translating the acceleration experienced by the animal's trunk into tag-dependent forces acting on the animal, with collars predicted to be particularly problematic. A tag that couples tightly with its carrier's trunk, such as one attached with tape to a bird [38] or glue to a marine mammal [39], experiences acceleration that closely matches that of its substrate, so it exerts forces at a site where most of the animal's mass lies. By contrast, a device on a looser-fitting collar of a moving tetrapod not only exerts forces on the (less massive) head and neck areas, rather than the animal's trunk, but the tag also oscillates between essentially two states: one is analogous to ‘freefall', which occurs between pulses of animal trunk acceleration in the stride cycle which project the collar in a particular direction owing to its inertia and lack of a tight couple with the neck. The collar is therefore subject to peaks in acceleration when it interacts with the animal's neck, causing greater collar acceleration than would be the case if it were tightly attached to the animal's body (cf. peaks in figure 1). This explains why Dickinson *et al*. [40] reported that acceleration signatures from collar-mounted tags deployed on (speed-controlled) goats *Capra aegagrus* became increasingly variable with increasing collar looseness, and is analogous to the concerns related to injuries sustained by people in vehicles depending on seatbelt tightness [41]. Partial answers to minimizing such problems may involve having padded collars that should reduce acceleration peaks, making sure that the tags themselves project minimally beyond the outer surface of the collar and having wider collars to reduce the pressure.

Having identified how animal movement changes the 3% tag rule, it is more problematic to understand how the identified forces translate into detriment. Within a general tag detriment framework, heavier tags require that animals perform more work (J) during movement because work done = force × distance, which helps clarify why the additional forces from a tag, on top of the animal weight, should relate to energy expenditure (cf. [42]). However, with respect to load carrying, how various tri-axial acceleration metrics such as DBA [26] relate to force and energy needs further research [43]. A prime effect of vectorially summed acceleration is that higher associated forces (because mass is constant) and smaller contact areas will lead to higher pressure at the tag-animal interface because pressure = force/area. This can affect anything from fur/feather wear [44] to changing the underlying tissue [45] and, as would be predicted, is notably prominent in species wearing thin collars (e.g. howler monkeys *Alouatta palliatai*, where 31% of animals wearing ball-chain radio-collars constituting just 1.2% of their mass sustained severe damage extending into the subcutaneous neck tissue and muscle [12]). However, pressure-dependent detriment will also depend on the proportion and length of time to which an animal is exposed to excessive forces, with animals that spend large proportions of their time travelling, such as wild dogs, being particularly susceptible [46].

Perhaps more esoteric, is the extent to which the inertia of a variable force-exerting tag ‘distracts' its wearer, aside from the physical issues of load-bearing by animals, and in this context, peak forces per stride are liable to be critical. The tag mass as a percentage of carrier mass did not affect the gait-specific speeds selected by the domestic dogs in this study. However, it remains to be seen the extent to which a typical 30 kg cheetah wearing a collar that is 3% of its body mass, and therefore experiencing an additional force equivalent to up to 16 kg during every bound of a prey pursuit, might have its hunting capacity compromised. We note that the survival of such animals is believed to be especially sensitive to the proportion of successful hunts (cf. [47]), which calls for critical evaluation of performance between tag-wearing and unequipped animals, or animals equipped with tags of different masses (cf. [32]).

In the meantime, our suggested approach of setting tag mass limits based on the overall (corrected) forces being less than 3% of the gravitational force exerted on the animal's body for 95% of the active time should go some way to getting a more realistic assessment of the potential for detriment. Where researchers adopting this approach do not have appropriate acceleration data for their study animal, they could use a surrogate species, perhaps from an online database. Such a resource should define the length of time that study animals were equipped to derive the acceleration frequency distribution because animal activities (and therefore the acceleration signals associated with them) occur variously over time. For this, longer periods are obviously better, but a pragmatic approach might be to plot cumulative frequencies of the vectorial sum of the acceleration as a function of recording time to see how they change or tend towards a stable value as the monitoring period increases. In this, we note that seasonal variation in animal behaviours, such as occurs in rutting ungulates, have potential to affect the distribution substantially, emphasizing the importance of considering the context under which the data were gathered.

Importantly, we do not advocate the 3% rule as such, but recognize that it has been widely adopted and could serve as a useful starting point with which to consider tag detriment if calculated as we have suggested here. In this, cognizance should also be given to the extent of tag forces for periods above the 95% threshold because, where these are excessive, it may be appropriate to use a 99% threshold or higher to derive appropriate tag masses. Notably though, even 99% limits do not highlight the high tag forces developed during prey pursuits exhibited by the cheetah. We suggest that the solution to this lies in more detailed consideration of the animal's lifestyle; in particular, identifying survival-critical behaviours with exceptionally high accelerations. Such periods may persuade ethics bodies to raise their thresholds still further. Underpinning this will be ongoing miniaturization, where tags benefit from the sensor revolution in human wearables, which will undoubtedly percolate through to animal applications: advanced smart phones have greater than 10 sensors, along with significant memory and data transmission capabilities, and typically weigh 150–200 g or about 0.2% of average human body mass, although human wearables benefit from regular contact with charging systems while many wildlife tag applications are projected for long-term deployments (e.g. [48]) that either necessitate correspondingly large batteries or autonomous charging systems, both of which increase the mass of tags [49].

Finally, consideration of the acceleration-based forces generated by animal-attached tags does not cover all forms of detriment because other forces are at play, such as greater drag in swimming- and flying species (cf. [6]), and more esoteric elements, such as device colour, that affect animal behaviour [50]. However, our framework should take the current ‘one-size-fits-all' basic 3% rule into an arena where quantitative assessment of acceleration can be compared to the myriad of tag-influenced behaviours recognized by the community to link animal lifestyle to putative detriment. Most importantly, these considerations should give ethics bodies a more useful rule of thumb than is currently the case and enable us to develop systems that minimize force-based tag effects, to the benefit of both animals and the science that their studies underpin.

## Data accessibility

Data pertaining to this study are available from the Dryad Digital Repository: https://doi.org/10.5061/dryad.rjdfn2zbm [51].

## References

[1] Brown DD, Kays R, Wikelski M, Wilson R, Klimley AP. 2013 Observing the unwatchable through acceleration logging of animal behavior. Animal Biotelemetry **1**, 1-20.

[2] Kays R, Crofoot MC, Jetz W, Wikelski M. 2015 Terrestrial animal tracking as an eye on life and planet. Science **348**, aaa2478. (10.1126/science.aaa2478) 26068858

[3] Wilson AM, Lowe JC, Roskilly K, Hudson PE, Golabek KA, McNutt JW. 2013 Locomotion dynamics of hunting in wild cheetahs. Nature **498**, 185-189. (10.1038/nature12295) 23765495

[4] Block BA et al. 2011 Tracking apex marine predator movements in a dynamic ocean. Nature **475**, 86-90. (10.1038/nature10082) 21697831

[5] Wilson RP, McMahon CR. 2006 Measuring devices on wild animals: what constitutes acceptable practice? Front. Ecol. Environ. **4**, 147-154. (10.1890/1540-9295(2006)004[0147:MDOWAW]2.0.CO;2)

[6] Saraux C et al. 2011 Reliability of flipper-banded penguins as indicators of climate change. Nature **469**, 203-206. (10.1038/nature09630) 21228875

[7] Culik BM, Wilson RP, Bannasch R. 1993 Flipper-bands on penguins: what is the cost of a life-long commitment? Marine Ecol. Progress Series **98**, 209-241. (10.3354/meps098209)

[8] Rosen DA, Gerlinsky CG, Trites AW. 2017 Telemetry tags increase the costs of swimming in northern fur seals, *Callorhinus ursinus*. Marine Mammal Sci. **34**, 385-402. (10.1111/mms.12460)

[9] Kay WP et al. 2019 Minimizing the impact of biologging devices: using computational fluid dynamics for optimizing tag design and positioning. Methods Ecol. Evol. **10**, 1222-1233. (10.1111/2041-210X.13216)

[10] Murray DL, Fuller MR. 2000 A critical review of the effects of marking on the biology of vertebrates. In Research techniques in animal ecology: controversies and consequences (eds MC Pearl, LF Biotani), pp. 15-64. New York, NY: Columbia University Press.

[11] Stabach JA et al. 2020 Short-term effects of GPS collars on the activity, behavior, and adrenal response of scimitar-horned oryx (*Oryx dammah*). PLoS ONE **15**, e0221843. (10.1371/journal.pone.0221843) 32045413 PMC7012457

[12] Hopkins M, Milton K. 2016 Adverse effects of ball-chain radio-collars on female mantled howlers (*Alouatta palliata*) in Panama. Int. J. Primatol. **37**, 213-224. (10.1007/s10764-016-9896-y)

[13] Krausman PR, Bleich VC, Cain III JW, Stephenson TR, DeYoung DW, McGarth PW, Swift PK, Pierce B,M, Jansen BD. 2004 Neck lesions in ungulates from collars incorporating satellite technology. Wildlife Soc. Bull. 1973-2006 **32**, 987-991.

[14] Brooks C, Bonyongo C, Harris S. 2010 Effects of global positioning system collar weight on zebra behavior and location error. Wildlife Soc. **72**, 527-534.

[15] Rasiulis AL, Marco FB, Couturier S, Cote SD. 2014 The effect of radio-collar weight on survival of migratory caribou. J. Wildlife Manag. **78**, 953-956. (10.1002/jwmg.722)

[16] Kenward RE. 2000 A manual for wildlife radio tagging. New York, NY: Academic press.

[17] Brander RB, Cochran WW. 1969 Radio location telemetry. Washington, DC: The Wildlife Society.

[18] Gessaman JA, Nagy KA. 1988 Transmitter loads affect the flight speed and metabolism of homing pigeons. Condor **90**, 662-668. (10.2307/1368356)

[19] Thaxter CB et al. 2016 Contrasting effects of GPS device and harness attachment on adult survival of lesser black-backed gulls *Larus fascus* and great skuas *Stercorarius skua*. Ibis **158**, 279-290. (10.1111/ibi.12340)

[20] Bodey TW, Cleasby IR, Bell F, Parr N, Schultz A, Votier SC, Bearhop S. 2017 A phylogenetically controlled meta-analysis of biologging device effects on birds: deleterious effects and a call for more standardized reporting of study data. Methods Ecol. Evol. **9**, 945-955.

[21] Portugal SJ, White CR. 2018 Miniaturization of biologgers is not alleviating the 5% rule. Methods Ecol. Evol. **9**, 1662-1666. (10.1111/2041-210x.13013)

[22] Barron DG, Brawn JD, Weatherhead PJ. 2010 Meta-analysis of transmitter effects on avian behaviour and ecology. Methods Ecol. Evol. **1**, 180-187. (10.1111/j.2041-210X.2010.00013.x)

[23] Gleiss AC, Wilson RP, Shepard EL. 2011 Making overall dynamic body acceleration work: on the theory of acceleration as a proxy for energy expenditure. Methods Ecol. Evol. **2**, 23-33. (10.1111/j.2041-210X.2010.00057.x)

[24] Boutaayamou M et al. 2015 Development and validation of an accelerometer-based method for quantifying gait events. Med. Eng. Phys. **37**, 226-232. (10.1016/j.medengphy.2015.01.001) 25618221

[25] Dickenson MH, Farley CT, Full RJ, Koehl MAR, Kram R, Lehman S. 2000 How animals move: an integrative view. Science **288**, 100-106. (10.1126/science.288.5463.100) 10753108

[26] Wilson RP et al. 2020 Estimates for energy expenditure in free-living animals using acceleration proxies: a reappraisal. J. Anim. Ecol. **89**, 161-172. (10.1111/1365-2656.13040) 31173339 PMC7030956

[27] Qasem L, Cardew A, Wilson A, Griffiths I, Halsey LG, Shepard ELC, Gleiss AC, Wilson R. 2012 Tri-axial dynamic acceleration as a proxy for animal energy expenditure; should we be summing values or calculating the vector? PLoS ONE **7**, e31187. (10.1371/journal.pone.0031187) 22363576 PMC3281952

[28] R Core Team. 2020 R: a language and environment for statistical computing. Vienna, Austria: R Foundation for Statistical Computing.

[29] Dewhirst OP, Evans HK, Roskilly K, Harvey RJ, Hubel TY, Wilson AM. 2016 Improving the accuracy of estimates of animal path and travel distance using GPS drift-corrected dead reckoning. Ecol. Evol. **6**, 6210-6222. (10.1002/ece3.2359) 27648238 PMC5016644

[30] Wilson RP, Sala JE, Gomez-Laich A, Ciancio J, Quintana F. 2015 Pushed to the limit: food abundance determines tag-induced harm in penguins. Animal Welfare **24**, 37-44. (10.7120/09627286.24.1.037)

[31] Pakanen V, Ronka N, Leslie TR, Blomqvist D, Koivula K. 2020 Survival probability in a small shorebird decreases with the time an individual carries a tracking device. J. Avian Biol. **51**, e02555. (10.1111/jav.02555)

[32] Wilson RP, Grant WS, Duffy DC. 1986 Recording devices on free-ranging marine animals: does measurement affect foraging performance? Ecology **67**, 1091-1093. (10.2307/1939832)

[33] Wu X, Pei B, Pei Y, Wu N, Zhou K, Hao Y, Wang W. 2019 Contributions of limb joints to energy absorption during landing in cats. Appl. Bionics Biomech **2019**, 3815612. (10.1155/2019/3815612) 31531125 PMC6721424

[34] Biewener AA. 2006 Patterns of mechanical energy change in tetrapod gait: pendula, springs and work. J. Exp. Zool. A **305**, 899-911. (10.1002/jez.a.334) 17029267

[35] Gregor RJ, Edgerton VR, Perrine JJ, Campion DS, DeBus C. 1979 Torque-velocity relationships and muscle fiber composition in elite female athletes. J. Appl. Physiol. **47**, 388-392. (10.1152/jappl.1979.47.2.388) 468696

[36] Alexander RM. 2002 Tendon elasticity and muscle function. Comp. Biochem. Physiol. A: Mol. Integr. Physiol. **133**, 1001-1011. (10.1016/S1095-6433(02)00143-5) 12485689

[37] Farley CT, Glasheen J, McMahon TA. 1993 Running springs: speed and animal size. J. Exp. Biol. **185**, 71-86. (10.1242/jeb.185.1.71) 8294853

[38] Wilson RP, Pütz K, Peters G, Culik B, Scolaro JA, Charrassin J-B, Ropert-Coudert Y. 1997 Long-term attachment of transmitting and recording devices to penguins and other seabirds. Wildlife Soc. Bull. (1973–2006) **25**, 101-106.

[39] Field IC, Harcourt RG, Boehme L, Bruyn PND, Charrassin JB, McMahon CR, Bester MN, Fedak MA, Hindell MA. 2012 Refining instrument attachment on phocid seals. Marine Mammal Sci. **28**, E325-E332. (10.1111/j.1748-7692.2011.00519.x)

[40] Dickinson ER, Stephens PA, Marks NJ, Wilson RP, Scantlebury DM. 2020 Best practice for collar deployment of tri-axial accelerometers on a terrestrial quadruped to provide accurate measurement of body acceleration. Animal Biotelem. **8**, 9. (10.1186/s40317-020-00198-9)

[41] Hodson-Walker N. 1970 The value of safety belts: a review. Can. Med. Assoc. J. **102**, 391. 4905861 PMC1946486

[42] Holewijn M. 1990 Physiological strain due to load carrying. Eur. J. Appl. Physiol. Occup. Physiol. **61**, 237-245. (10.1007/BF00357606) 2282907

[43] Tikkanen O, Karkkainen S, Haakana P, Kallinen M, Pullinen T, Finni T. 2014 EMG, heart rate, and accelerometer as estimators of energy expenditure in locomotion. Med. Sci. Sports Exerc. **46**, 1831-1839. (10.1249/MSS.0000000000000298) 24504428

[44] Young VKH, Blob RW. 2016 Comparative limb bone scaling and shape in turtles: relationships with functional demands. Integr. Comp. Biol. **56**, E398.

[45] Michael S, Gartrell B, Hunter S. 2013 Humeral remodeling and soft tissue injury of the wings caused by backpack harnesses for radio transmitters in New Zealand takahe (*Porphyrio hochstetteri*). J. Wildl Dis. **49**, 552-559. (10.7589/2013-1-006) 23778604

[46] Pomilia MA, McNutt JW, Jorndan MR. 2015 Ecological predictors of African wild dog ranging patterns in northern Botswana. J. Mammalogy **96**, 1214-1223.

[47] Scantlebury DM et al. 2014 Flexible energetics of cheetah hunting strategies provide resistance against kleptoparasitism. Science **346**, 79-81. (10.1126/science.1256424) 25278609

[48] Pennisi E. 2011 Animal ecology. Global tracking of small animals gains momentum. Science **334**, 1042. (10.1126/science.334.6059.1042) 22116848

[49] Holton MD, Wilson RP, Teilmann J, Siebert U. 2021 Animal tag technology keeps coming of age: an engineering perspective. Phil. Trans. R. Soc. Lond. B **376**, 20200229. (10.1098/rstb.2020.0229) 34176328 PMC8237169

[50] Wilson RP, Spairani HJ, Coria NR, Culik BM, Adelung D. 1990 Packages for attachment to seabirds - what color do adelie penguins dislike least. J. Wildlife Manage. **54**, 447-451. (10.2307/3809657)

[51] Wilson RP et al. 2021 Data from: Animal lifestyle affects acceptable mass limits for attached tags. *Dryad Digital Repository*. (10.5061/dryad.rjdfn2zbm) PMC8548787 34702077

