# Correction to ‘Animal lifestyle affects acceptable mass limits for attached tags’

**DOI:** 10.1098/rspb.2022.0120

**Published:** 2022-02-09

**Authors:** Rory P. Wilson, Kayleigh A. Rose, Richard Gunner, Mark D. Holton, Nikki J. Marks, Nigel C. Bennett, Stephen H. Bell, Joshua P. Twining, Jamie Hesketh, Carlos M. Duarte, Neil Bezodis, Milos Jezek, Michael Painter, Vaclav Silovsky, Margaret C. Crofoot, Roi Harel, John P. Y. Arnould, Blake M. Allan, Desley A. Whisson, Abdulaziz Alagaili, D. Michael Scantlebury

*Proc. R. Soc. B* **288**, 20212005. Published online 27 October 2021. (doi:10.1098/rspb.2021.2005)

Correction details:

Author Rory P. Wilson's affiliations should also include the Max Planck Institute of Animal Behavior:

Swansea Laboratory for Animal Movement, Biosciences, College of Science, Swansea University, Singleton Park, Swansea SA2 8PP, UK

Max Planck Institute of Animal Behavior, 78315 Radolfzell, Germany

