# Supplementary material for: Animal lifestyle affects acceptable mass limits for attached tags
Source: Proc Biol Sci. 2021 Oct 27;288(1961):20212005. doi: 10.1098/rspb.2021.2005 (PMC8548787; doi:10.1098/rspb.2021.2005)
Supplement: Supplementary file 1 [file rspb20212005_review_history.pdf]

THE ROYAL SOCIETY
PUBLISHING

PROCEEDINGS B

Animal lifestyle affects acceptable mass limits for attached tags

Rory P. Wilson, Kayleigh A. Rose, Richard Gunner, Mark D. Holton, Nikki J. Marks, Nigel C. Bennett, Stephen H. Bell, Joshua P. Twining, Jamie Hesketh, Carlos M. Duarte, Neil Bezodis, Milos Jezek, Michael Painter, Vaclav Silovsky, Margaret C. Crofoot, Roi Harel, John P. Y. Arnould, Blake M. Allan, Desley A. Whisson, Abdulaziz Alagaili and D. Michael Scantlebury

Article citation details
Proc. R. Soc. B **288**: 20212005.
http://dx.doi.org/10.1098/rspb.2021.2005

Review timeline

Original submission:	6 June 2021
1st revised submission:	9 September 2021
2nd revised submission:	4 October 2021
Final acceptance:	5 October 2021

Note: Reports are unedited and appear as submitted by the referee. The review history appears in chronological order.

Review History

RSPB-2021-1207.R0 (Original submission)

Review form: Reviewer 1 (Anthony Pagano)

Recommendation
Accept with minor revision (please list in comments)

Scientific importance: Is the manuscript an original and important contribution to its field?
Excellent

General interest: Is the paper of sufficient general interest?
Good

Quality of the paper: Is the overall quality of the paper suitable?
Good

Is the length of the paper justified?
Yes

Reports © 2021 The Reviewers; Decision Letters © 2021 The Reviewers and Editors; Responses © 2021 The Reviewers, Editors and Authors. Published by the Royal Society under the terms of the Creative Commons Attribution License http://creativecommons.org/licenses/by/4.0/, which permits unrestricted use, provided the original author and source are credited

Should the paper be seen by a specialist statistical reviewer?
No

Do you have any concerns about statistical analyses in this paper? If so, please specify them explicitly in your report.
No

It is a condition of publication that authors make their supporting data, code and materials available - either as supplementary material or hosted in an external repository. Please rate, if applicable, the supporting data on the following criteria.

Is it accessible?
Yes

Is it clear?
Yes

Is it adequate?
No

Do you have any ethical concerns with this paper?
No

Comments to the Author
This manuscript presents a novel approach for evaluating the force imposed by animal-attached devices. As such, it addresses an important topic regarding the appropriate mass of these devices and should be of broad interest to ecologists and wildlife biologists. In particular, the authors highlight an important point that animal athleticism and behavior should be considered when evaluating tag suitability in addition to body mass. Overall, the manuscript is well written and the analyses are suitable in addressing the research topic.

My comments below focus on clarifying details of the manuscript that are unclear in some areas:

Abstract

Lines 57 – 58: These analyses all relate to data from carnivores. That should be clarified here.

Introduction

Lines 100 – 100: I recommend the authors reference Brander and Cochran (1969) here. This reference provides an approximate 5% justification under the criteria that the weight of the tag does not cause an apparent change in behavior patterns. Additionally, I recommend the authors expand on this statement. There are a wealth of studies that have reviewed whether the 3 – 5% rule is appropriate.

Lines 110 – 121: The ordering here is confusing as it is unclear how the non-carnivore data is being used. I recommend either combining the carnivores and non-carnivores in this text or separately explaining how the data from the non-carnivores is used in comparison to the data from the carnivores and domestic dogs.

Materials and Methods

Line 139: It would be useful to know the weights of these collars, mean and range. Additionally, a Supplemental table listing each species and the collar mass used and its percent mass relative to the species body mass is needed.

Lines 146 – 149: Provide the mean mass of each species as was done for the carnivores.

Lines 146 – 149: This is redundant with the introduction. I recommend shortening this to just a list of the species rather than listing the Family names again.

Line 149: It would again be useful to know the weights of these collars, mean and range.

Lines 178 – 181: I believe all species (including the non-carnivores) had raw acceleration converted to the vectorial sum. It would be preferable to indicate that here.

Lines 178 – 181: Typically, dynamic acceleration (ODBA or VeDBA) is used rather than raw acceleration that includes the effects of gravity. It would be helpful for the authors to explain why they have chosen to use raw acceleration rather than dynamic acceleration.

Results

Lines 225 – 227: The significance of acceleration exceeding 1 g in that it will result in force exceeding 3% (assuming a 3% tag mass) should be explained in the introduction or methods.

Lines 225 – 227: I believe this refers to the percentage time during each specific behavior (walk, trot, bound). If so, that should be clarified here and in the table heading to Table S1.

Line 230: I believe this should refer to Figure 2 not Figure 1.

Lines 256 – 257: This sentence is confusing at the start of the paragraph. Suggest rewording.

Lines 259 – 261: Why do the authors assume the tags amounted to 3% of their body mass rather than using the actual mass of the collars that were deployed? As I mention in my comments for lines 354 – 358, a tag (collar) that is 3% of the mass of a 180 kg lion seems unlikely to be deployed.

Figure 3: Again, I question whether it is reasonable to assume a tag that is 3% of the body mass of a lion. It seems unlikely that such a heavy tag (collar) would be deployed regardless of the 3 – 5% justification. It would be informative to evaluate how these results would compare using a typical collar mass for a lion (e.g., ~1.2 kg) as well as the 3% mass the authors present here.

Figure 5A: The blue arrows and species labels in this figure could be removed. I found them to be confusing particularly since the Marten arrow is pointing at the Koala line.

Figure 5B: I suggest labeling the Z axis as "Derived tag mass as percentage of body mass". Currently it is not apparent that this value is derived from the corresponding measure of force.

Lines 300 – 303: For future researchers to apply these methods, it would be helpful to describe how the authors determined when animals were active versus inactive.

Discussion

Lines 346 – 347: Again, the significance of tag forces > 1 g should be explained prior to this.

Lines 348 – 351: It would be good to qualify that the 3-5% mass limits seem appropriate for slow-moving animals based on the measure of force, which does not mean such tags may not nevertheless influence these animals' behavior.

Lines 354 – 358: Though a tag 3% of the mass of a lion seems unlikely (i.e. 5.4 kg). In extremely large animals the 3% rule is less likely to be a limiting factor. Whereas in smaller species it is more likely to influence tag restrictions. It would be worth noting this point in the discussion.

Line 356: Do the authors' findings suggest that energy expenditure estimates derived from acceleration may be impacted by the weight of the tag? How does the tag driven force influence DBA measurements?

Lines 360 – 379: These are important points. Did the authors consider testing this in their dog trials (comparing the forces of mounted tags versus collar mounted tags)?

Lines 404 – 406: It would be helpful to clarify here that the "95% of the time" refers to "95% of the time while active".

Lines 425 – 427: This discussion ignores the issue of battery life and weight, which is often the heaviest component of wildlife tags. Human wearables are able to use rechargeable batteries that may last for hours to days before needing to be recharged. Such recharging options are typically not available or suitable for wildlife applications. I recommend highlighting the need for either more energy efficient sensors and/or advances in battery performance and longevity.

Supplementary Materials

Lines 25 – 30: The main text indicates data were collected during normal activities in the wild. It's unclear whether this cheetah data was collected in the wild?

Line 54: Please provide details on what type of collars these were.

Lines 67 – 70: Details on pine martins are provided on lines 15 – 23. I believe these lines can be deleted.

Line 80: Provide details on what type of collars these were.

Line 121: Less than 80 g is vague. Can the authors provide a more precise value here or provide a range?

Data Release

I do not believe the data and analysis code for the full cross-species comparison are provided. The cumulative frequency curves of the vectoral sum of the acceleration will have the greatest relevance to future researchers wishing to apply these methods. Hence, it would be useful if these data and analysis code are included.

Review form: Reviewer 2

Recommendation
Reject – article is not of sufficient interest (we will consider a transfer to another journal)

Scientific importance: Is the manuscript an original and important contribution to its field?
Acceptable

General interest: Is the paper of sufficient general interest?
Acceptable

Quality of the paper: Is the overall quality of the paper suitable?
Acceptable

Is the length of the paper justified?
Yes

Should the paper be seen by a specialist statistical reviewer?
No

Do you have any concerns about statistical analyses in this paper? If so, please specify them explicitly in your report.
No

It is a condition of publication that authors make their supporting data, code and materials available - either as supplementary material or hosted in an external repository. Please rate, if applicable, the supporting data on the following criteria.

Is it accessible?
Yes

Is it clear?
Yes

Is it adequate?
No

Do you have any ethical concerns with this paper?
No

Comments to the Author
This is an interesting well-written paper addressing an important issue. The approach to biologging certainly does need this kind of thinking. I do wonder if it would be better suited to Methods in Ecology and Evolution.

Major Comments

1. There is potential to me that the whole framework of the paper is a circular argument. If the accelerations are being taken off the tags when on animals, how do you know that the devices weren't already impacting acceleration? I.e., your baselies are not realistic baselines.
2. Species chosen – I think more rationale needs to be added here. At the moment, it reads like a slightly random list of animals, that likely were down to availability and ease of access. That is fine, in a way, but really needs a better argument. The rationale presented is incredibly brief, and doesn't really fully define the life history of the species.
3. Sample sizes – some are incredibly small (N = 1). Should a study really be advocating an entirely new approach to decision making when it comes to biologging (which I fully support) on such small sample sizes?
4. Dog gaits – what criteria did you use to visually inspect gaits? This seems somewhat superficial. These need clear definitions, and how many observers decided on these gaits just using their eyes?
5. Gait – in the Introduction and Methods, gait is now a major point, but then in the Results, is given much prominence. This leads, as a reader, to an element of mis-match.

Minor Comments

- Latins need adding for certain species.
- Line 78: I think smart tag is a specific term? Either needs to be defined or just use biologger.
- Line 85: Can you be more specific about what the effect on the penguin populations was? A reduction one assumes? 1%? 85%
- Line 157: Is there an issue with the owners providing body mass of their dogs? Potential for bias? Heavily inaccurate scales?

- Line 220: "most likely corresponded" - I think this needs to be somewhat more stringent?
- Discussion – a random opening, given the theme of the paper. A useful point to make, but feel the Discussion would work better opening with some confirmation of the primary results.
- Some minor referencing issues.

Review form: Reviewer 3 (Jasper Eikelboom)

Recommendation
Major revision is needed (please make suggestions in comments)

Scientific importance: Is the manuscript an original and important contribution to its field?
Excellent

General interest: Is the paper of sufficient general interest?
Excellent

Quality of the paper: Is the overall quality of the paper suitable?
Good

Is the length of the paper justified?
Yes

Should the paper be seen by a specialist statistical reviewer?
No

Do you have any concerns about statistical analyses in this paper? If so, please specify them explicitly in your report.
No

It is a condition of publication that authors make their supporting data, code and materials available - either as supplementary material or hosted in an external repository. Please rate, if applicable, the supporting data on the following criteria.

> **Is it accessible?**
> Yes
>
> **Is it clear?**
> Yes
>
> **Is it adequate?**
> No

Do you have any ethical concerns with this paper?
Yes

Comments to the Author
Main comments:
I applaud the aim and execution of this paper to scrutinize the (largely unsubstantiated) rule-of-thumb used for animal-born tag mass. I think the presented cumulative frequency graphs and the proposed method for determining maximum tag masses based on g-forces exerted on animals are interpretable and appropriate, which could lead to a quick change in guidelines and practices

used by animal researchers and ethical committees.

However, my main concern with the presented method is the focus on the 95% lowest accelerations as a limit for tag mass, where (as the authors already mention themselves in L396) it are often the periods with the most extreme g-forces that impact animal the most (either through direct strain of the forces exerted on the body or through indirect effects by e.g. impacting hunting success). Shifting the focus of the method to the upper range of the g-forces would therefore do more justice to these considerations, for which I make suggestions below.

Specific comments:
L2: "changes" may at first be read as a noun instead of a verb. Perhaps "affects" or "influences" is better.
L79: "scales" is multi-interpretable in this context.
L101: This undermines the recommendation in the abstract of targeting <3% body weight in force for 95% of the time. Why should we keep adhering to this 3% rule?
L138: Please also provide the sensitivity or resolution of the accelerometer, so that we can get an impression of the granularity of the data (as 0-32g is quite a large range, the data could probably be quite granular, which may impact the accurate determination of the force belonging to 3% body weight).
L140: For me it is not clear if all components attached to the collar were in the same spot as the accelerometer. Is there for example not a counterweight on the other side of the collar to prevent rotation? Or the battery at the other end of the collar compared to the sensor case? This setup is critical to know for the conversion to forces on the animal body.
L144-149: This is almost a copy of the paragraph in the introduction, I would remove one of them.
L149: I would prefer to know the weight distribution of the collars of these animals in the main text as well (just as for the carnivores and the dogs), as this is important information for the reader as to how they should compare the results for these different groups.
L162: So the load was spread equally along the collar ("along their full-length")? Please make this 100% clear in the text, because to me this contradicts with "stacked" (perhaps because I'm not a native speaker, but many people aren't). If it's equally spread, then it does however differ from the situation with the carnivores where the load was positioned (presumably?) at one location on the collar.
L164: Given that it's the same accelerometer, it is not needed to give all specs again.
L170: Not "gallop" or "canter"? "Bound" is used almost everywhere in the text, but once "gallop" is used as well. I would check which name is used more often in the literature for this gait.
L187: But m is the mass of the entire collar, not of the sensor, right? If m is distributed equally along a collar then the acceleration of the sensor does not apply to the whole mass in the same way.
L198: Please give R version instead of RStudio version. Also lme4 version, as those two versions primarily determine the functioning of the code.
L201: Why isn't collar mass a covariate? Even though there are only a few categories, there certainly is an order in these (0<1<2<3).
L209: Reported where?
L218-220: Determined how?
L221-222: What is meant with the text about dogs here?
L223: So for clarification: the cumulative frequency plots did not use the peak accelerations? To quantify strain on the animal, I would be inclined to make frequency plots solely of these peak acceleration values instead of all acceleration values. This has the benefit that it focuses on the maximum force of each cyclic period, thereby giving more attention in the method to the highest forces exerted by the tag (which ultimately determines the effect on the animal the most, not the 95% lowest forces). Furthermore, based on our own experiences we can state that for each step that we take (which these acceleration periods mostly are) the forces that impact us the most are the maximum values, e.g., when our feet hit the ground during running. Or compared to horse riding: it are the peak downward forces of the rider on the horse's back during trotting that determine the strain of the saddle on the horse. So why take all the lower forces also into account in the frequency plots when these do not really matter for the animal? Furthermore, it makes the

analysis more coherent when it is centred all around these peak accelerations, because currently the analyses considering the peak acceleration in carnivores and dogs are somewhat disjunct from the cumulative frequency plots.
L263: What does the x-axis mean? Shouldn't it be "time since start of chase"?
L268: Why do the trend lines continue before 0% and after 100%?
L276: Why are there two models specified in Table S4 (A and D) for peak acceleration? A also has non-significant variables included.
L280: But the this moving object did not have this 1-3% mass, right? So even though the movement was exacerbated, the force exerted on the animal's body presumably not (as it rotated along the neck, instead of pounding in one place).
L284-286: Why isn't the intercept fixed? With a speed of 0, the vectorial sum g-force is 1 (gravity), so you can fix the intercept at 1. This reduced the degrees of freedom of the model fit and allows for a more straight-forward comparison of the slopes of the different tags.
L399-402: I fully agree, but I posit this paper can already contribute more to this problem by acknowledging that the peak accelerations matter the most. Or alternatively in some other way provide a measure that focuses more on the highest g-forces exerted on the animal. In the currently proposed 3/95%-threshold method it does not matter how high the g-forces become after the 95% threshold, but the method of constructing the cumulative frequency graph does allow for it. Only focussing on the amount of time where the tag exerts more than 3% body weight in force reduces the dimensionality of the strain quite a lot.
L413: Most readers will solely read the abstract and act according to that, so they will assume the authors do advocate for this 3/95% rule. Stating here that the authors do not advocate the 3% rule does not seem to match with the importance of the 3% rule in the method and the prominent place it has in the abstract. In the abstract it is even stated that 3/95% is a "scientifically robust acceptable threshold", but currently I haven't see a scientific justification for both the 3% and the 95% rule. I do think the cumulative frequency plots can provide more metrics that are also relevant for tag detriment (focusing on the upper range of values), which goes beyond the 3/95% rule. This paragraph already describes the importance of doing this, so I'd encourage the authors to showcase this practice in the current study (as all the ingredients for doing it are there) by liking it more to the visualization in Fig. 3 about the more extreme Gs and/or providing quantitative metrics that are also based on the cumulative frequency plots that describe tag detriment from the upper range of acceleration values (e.g., skewness, weighted area under the curve, etc).

Decision letter (RSPB-2021-1207.R0)

27-Jul-2021

Dear Dr Wilson:

I am writing to inform you that your manuscript RSPB-2021-1207 entitled "Animal lifestyle changes acceptable mass limits for attached tags" has, in its current form, been rejected for publication in Proceedings B.

This action has been taken on the advice of referees, who have recommended that substantial revisions are necessary. With this in mind we would be happy to consider a resubmission, provided the comments of the referees are fully addressed. However please note that this is not a provisional acceptance.

The resubmission will be treated as a new manuscript. However, we will approach the same reviewers if they are available and it is deemed appropriate to do so by the Editor. Please note that resubmissions must be submitted within six months of the date of this email. In exceptional

circumstances, extensions may be possible if agreed with the Editorial Office. Manuscripts submitted after this date will be automatically rejected.

Please find below the comments made by the referees, not including confidential reports to the Editor, which I hope you will find useful. If you do choose to resubmit your manuscript, please upload the following:

1) A ‘response to referees’ document including details of how you have responded to the comments, and the adjustments you have made.
2) A clean copy of the manuscript and one with 'tracked changes' indicating your 'response to referees' comments document.
3) Line numbers in your main document.
4) Data - please see our policies on data sharing to ensure that you are complying (https://royalsociety.org/journals/authors/author-guidelines/#data).

**Note that the data/code were deemed insufficient in review-- Comments were made that: "It only contains summarized data and scripts that are used for the linear models. The raw data and code is missing from which the peak accelerations and cumulative frequency plots are constructed that are presented in the paper." and "The data releases are clear and well annotated. However, I do not believe the data and analysis code for the full cross-species comparison are provided. Only the data and analysis code for the 4 species of free-living carnivores and domestic dogs appear to be available. The cumulative frequency curves of the vectoral sum of the acceleration will have the greatest relevance to future researchers wishing to apply these methods. Hence, it would be useful if these data and analysis code are included." All 3 reviewers raised concerns on this topic and it must be fully addressed in revision.

To upload a resubmitted manuscript, log into http://mc.manuscriptcentral.com/prsb and enter your Author Centre, where you will find your manuscript title listed under "Manuscripts with Decisions." Under "Actions," click on "Create a Resubmission." Please be sure to indicate in your cover letter that it is a resubmission, and supply the previous reference number.

Sincerely,
Dr John Hutchinson, Editor
mailto: proceedingsb@royalsociety.org

Associate Editor
Board Member: 1
Comments to Author:
This paper deals with an important topic in animal research and seeks to influence future policy on animal experimentation that could have important impacts on welfare and scientific practice across a range of biological disciplines. It is therefore no surprise that the reviewers are supportive of the aims and the potential significance of such a study. However, the reviewers vary in their assessment of the quality and robustness of how the current study was carried out. Reviewer 1 provides detailed editorial recommendations that will improve the clarity and discussion currently presented in the manuscript. Reviewer 3 provides editorial suggestions, but also questions whether the specific metrics extracted from the collected data are the most appropriate for the study goals. Reviewer 2 is more critical and questions more fundamental aspects of the study such as its design and sample size. My feeling is that nearly all the comments made by the reviewers require very careful consideration, which will likely result in some re-analysis and potentially collection of some additional data. For example, it might be possible to address a number of the concerns raised by reviewer 1 through analysis of new/additional data from a small sample of domestic dogs. As a further point, the reviewers note that not all raw data required to repeat the analyses were available with the first submission, and that ethical approval (i.e. by an ethics committee) for the lion study was not stated.

Reviewer(s)' Comments to Author:
Referee: 1
Comments to the Author(s)
This manuscript presents a novel approach for evaluating the force imposed by animal-attached devices. As such, it addresses an important topic regarding the appropriate mass of these devices and should be of broad interest to ecologists and wildlife biologists. In particular, the authors highlight an important point that animal athleticism and behavior should be considered when evaluating tag suitability in addition to body mass. Overall, the manuscript is well written and the analyses are suitable in addressing the research topic.

My comments below focus on clarifying details of the manuscript that are unclear in some areas:

Abstract

Lines 57 – 58: These analyses all relate to data from carnivores. That should be clarified here.

Introduction

Lines 100 – 100: I recommend the authors reference Brander and Cochran (1969) here. This reference provides an approximate 5% justification under the criteria that the weight of the tag does not cause an apparent change in behavior patterns. Additionally, I recommend the authors expand on this statement. There are a wealth of studies that have reviewed whether the 3 – 5% rule is appropriate.

Lines 110 – 121: The ordering here is confusing as it is unclear how the non-carnivore data is being used. I recommend either combining the carnivores and non-carnivores in this text or separately explaining how the data from the non-carnivores is used in comparison to the data from the carnivores and domestic dogs.

Materials and Methods

Line 139: It would be useful to know the weights of these collars, mean and range. Additionally, a Supplemental table listing each species and the collar mass used and its percent mass relative to the species body mass is needed.

Lines 146 – 149: Provide the mean mass of each species as was done for the carnivores.

Lines 146 – 149: This is redundant with the introduction. I recommend shortening this to just a list of the species rather than listing the Family names again.

Line 149: It would again be useful to know the weights of these collars, mean and range.

Lines 178 – 181: I believe all species (including the non-carnivores) had raw acceleration converted to the vectorial sum. It would be preferable to indicate that here.

Lines 178 – 181: Typically, dynamic acceleration (ODBA or VeDBA) is used rather than raw acceleration that includes the effects of gravity. It would be helpful for the authors to explain why they have chosen to use raw acceleration rather than dynamic acceleration.

Results

Lines 225 – 227: The significance of acceleration exceeding 1 g in that it will result in force exceeding 3% (assuming a 3% tag mass) should be explained in the introduction or methods.

Lines 225 – 227: I believe this refers to the percentage time during each specific behavior (walk, trot, bound). If so, that should be clarified here and in the table heading to Table S1.

Line 230: I believe this should refer to Figure 2 not Figure 1.

Lines 256 – 257: This sentence is confusing at the start of the paragraph. Suggest rewording.

Lines 259 – 261: Why do the authors assume the tags amounted to 3% of their body mass rather than using the actual mass of the collars that were deployed? As I mention in my comments for lines 354 – 358, a tag (collar) that is 3% of the mass of a 180 kg lion seems unlikely to be deployed.

Figure 3: Again, I question whether it is reasonable to assume a tag that is 3% of the body mass of a lion. It seems unlikely that such a heavy tag (collar) would be deployed regardless of the 3 – 5% justification. It would be informative to evaluate how these results would compare using a typical collar mass for a lion (e.g., ~1.2 kg) as well as the 3% mass the authors present here.

Figure 5A: The blue arrows and species labels in this figure could be removed. I found them to be confusing particularly since the Marten arrow is pointing at the Koala line.

Figure 5B: I suggest labeling the Z axis as "Derived tag mass as percentage of body mass". Currently it is not apparent that this value is derived from the corresponding measure of force.

Lines 300 – 303: For future researchers to apply these methods, it would be helpful to describe how the authors determined when animals were active versus inactive.

Discussion

Lines 346 – 347: Again, the significance of tag forces > 1 g should be explained prior to this.

Lines 348 – 351: It would be good to qualify that the 3-5% mass limits seem appropriate for slow-moving animals based on the measure of force, which does not mean such tags may not nevertheless influence these animals' behavior.

Lines 354 – 358: Though a tag 3% of the mass of a lion seems unlikely (i.e. 5.4 kg). In extremely large animals the 3% rule is less likely to be a limiting factor. Whereas in smaller species it is more likely to influence tag restrictions. It would be worth noting this point in the discussion.

Line 356: Do the authors' findings suggest that energy expenditure estimates derived from acceleration may be impacted by the weight of the tag? How does the tag driven force influence DBA measurements?

Lines 360 – 379: These are important points. Did the authors consider testing this in their dog trials (comparing the forces of mounted tags versus collar mounted tags)?

Lines 404 – 406: It would be helpful to clarify here that the "95% of the time" refers to "95% of the time while active".

Lines 425 – 427: This discussion ignores the issue of battery life and weight, which is often the heaviest component of wildlife tags. Human wearables are able to use rechargeable batteries that may last for hours to days before needing to be recharged. Such recharging options are typically not available or suitable for wildlife applications. I recommend highlighting the need for either more energy efficient sensors and/or advances in battery performance and longevity.

Supplementary Materials

Lines 25 – 30: The main text indicates data were collected during normal activities in the wild. It's unclear whether this cheetah data was collected in the wild?

Line 54: Please provide details on what type of collars these were.

Lines 67 – 70: Details on pine martins are provided on lines 15 – 23. I believe these lines can be deleted.

Line 80: Provide details on what type of collars these were.

Line 121: Less than 80 g is vague. Can the authors provide a more precise value here or provide a range?

Data Release

I do not believe the data and analysis code for the full cross-species comparison are provided. The cumulative frequency curves of the vectoral sum of the acceleration will have the greatest relevance to future researchers wishing to apply these methods. Hence, it would be useful if these data and analysis code are included.

Referee: 2
Comments to the Author(s)
This is an interesting well-written paper addressing an important issue. The approach to biologging certainly does need this kind of thinking. I do wonder if it would be better suited to Methods in Ecology and Evolution.

Major Comments

1. There is potential to me that the whole framework of the paper is a circular argument. If the accelerations are being taken off the tags when on animals, how do you know that the devices weren't already impacting acceleration? I.e., your baselies are not realistic baselines.
2. Species chosen – I think more rationale needs to be added here. At the moment, it reads like a slightly random list of animals, that likely were down to availability and ease of access. That is fine, in a way, but really needs a better argument. The rationale presented is incredibly brief, and doesn't really fully define the life history of the species.
3. Sample sizes – some are incredibly small (N = 1). Should a study really be advocating an entirely new approach to decision making when it comes to biologging (which I fully support) on such small sample sizes?
4. Dog gaits – what criteria did you use to visually inspect gaits? This seems somewhat superficial. These need clear definitions, and how many observers decided on these gaits just using their eyes?
5. Gait – in the Introduction and Methods, gait is now a major point, but then in the Results, is given much prominence. This leads, as a reader, to an element of mis-match.

Minor Comments

• Latins need adding for certain species.
• Line 78: I think smart tag is a specific term? Either needs to be defined or just use biologger.
• Line 85: Can you be more specific about what the effect on the penguin populations was? A reduction one assumes? 1%? 85%
• Line 157: Is there an issue with the owners providing body mass of their dogs? Potential for bias? Heavily inaccurate scales?
• Line 220: "most likely corresponded" - I think this needs to be somewhat more stringent?
• Discussion – a random opening, given the theme of the paper. A useful point to make, but feel the Discussion would work better opening with some confirmation of the primary results.

• Some minor referencing issues.

Referee: 3
Comments to the Author(s)
Main comments:
I applaud the aim and execution of this paper to scrutinize the (largely unsubstantiated) rule-of-thumb used for animal-born tag mass. I think the presented cumulative frequency graphs and the proposed method for determining maximum tag masses based on g-forces exerted on animals are interpretable and appropriate, which could lead to a quick change in guidelines and practices used by animal researchers and ethical committees.

However, my main concern with the presented method is the focus on the 95% lowest accelerations as a limit for tag mass, where (as the authors already mention themselves in L396) it are often the periods with the most extreme g-forces that impact animal the most (either through direct strain of the forces exerted on the body or through indirect effects by e.g. impacting hunting success). Shifting the focus of the method to the upper range of the g-forces would therefore do more justice to these considerations, for which I make suggestions below.

Specific comments:
L2: "changes" may at first be read as a noun instead of a verb. Perhaps "affects" or "influences" is better.
L79: "scales" is multi-interpretable in this context.
L101: This undermines the recommendation in the abstract of targeting <3% body weight in force for 95% of the time. Why should we keep adhering to this 3% rule?
L138: Please also provide the sensitivity or resolution of the accelerometer, so that we can get an impression of the granularity of the data (as 0-32g is quite a large range, the data could probably be quite granular, which may impact the accurate determination of the force belonging to 3% body weight).
L140: For me it is not clear if all components attached to the collar were in the same spot as the accelerometer. Is there for example not a counterweight on the other side of the collar to prevent rotation? Or the battery at the other end of the collar compared to the sensor case? This setup is critical to know for the conversion to forces on the animal body.
L144-149: This is almost a copy of the paragraph in the introduction, I would remove one of them.
L149: I would prefer to know the weight distribution of the collars of these animals in the main text as well (just as for the carnivores and the dogs), as this is important information for the reader as to how they should compare the results for these different groups.
L162: So the load was spread equally along the collar ("along their full-length")? Please make this 100% clear in the text, because to me this contradicts with "stacked" (perhaps because I'm not a native speaker, but many people aren't). If it's equally spread, then it does however differ from the situation with the carnivores where the load was positioned (presumably?) at one location on the collar.
L164: Given that it's the same accelerometer, it is not needed to give all specs again.
L170: Not "gallop" or "canter"? "Bound" is used almost everywhere in the text, but once "gallop" is used as well. I would check which name is used more often in the literature for this gait.
L187: But m is the mass of the entire collar, not of the sensor, right? If m is distributed equally along a collar then the acceleration of the sensor does not apply to the whole mass in the same way.
L198: Please give R version instead of RStudio version. Also lme4 version, as those two versions primarily determine the functioning of the code.
L201: Why isn't collar mass a covariate? Even though there are only a few categories, there certainly is an order in these (0<1<2<3).
L209: Reported where?
L218-220: Determined how?
L221-222: What is meant with the text about dogs here?

L223: So for clarification: the cumulative frequency plots did not use the peak accelerations? To quantify strain on the animal, I would be inclined to make frequency plots solely of these peak acceleration values instead of all acceleration values. This has the benefit that it focuses on the maximum force of each cyclic period, thereby giving more attention in the method to the highest forces exerted by the tag (which ultimately determines the effect on the animal the most, not the 95% lowest forces). Furthermore, based on our own experiences we can state that for each step that we take (which these acceleration periods mostly are) the forces that impact us the most are the maximum values, e.g., when our feet hit the ground during running. Or compared to horse riding: it are the peak downward forces of the rider on the horse's back during trotting that determine the strain of the saddle on the horse. So why take all the lower forces also into account in the frequency plots when these do not really matter for the animal? Furthermore, it makes the analysis more coherent when it is centred all around these peak accelerations, because currently the analyses considering the peak acceleration in carnivores and dogs are somewhat disjunct from the cumulative frequency plots.
L263: What does the x-axis mean? Shouldn't it be "time since start of chase"?
L268: Why do the trend lines continue before 0% and after 100%?
L276: Why are there two models specified in Table S4 (A and D) for peak acceleration? A also has non-significant variables included.
L280: But the this moving object did not have this 1-3% mass, right? So even though the movement was exacerbated, the force exerted on the animal's body presumably not (as it rotated along the neck, instead of pounding in one place).
L284-286: Why isn't the intercept fixed? With a speed of 0, the vectorial sum g-force is 1 (gravity), so you can fix the intercept at 1. This reduced the degrees of freedom of the model fit and allows for a more straight-forward comparison of the slopes of the different tags.
L399-402: I fully agree, but I posit this paper can already contribute more to this problem by acknowledging that the peak accelerations matter the most. Or alternatively in some other way provide a measure that focuses more on the highest g-forces exerted on the animal. In the currently proposed 3/95%-threshold method it does not matter how high the g-forces become after the 95% threshold, but the method of constructing the cumulative frequency graph does allow for it. Only focussing on the amount of time where the tag exerts more than 3% body weight in force reduces the dimensionality of the strain quite a lot.
L413: Most readers will solely read the abstract and act according to that, so they will assume the authors do advocate for this 3/95% rule. Stating here that the authors do not advocate the 3% rule does not seem to match with the importance of the 3% rule in the method and the prominent place it has in the abstract. In the abstract it is even stated that 3/95% is a "scientifically robust acceptable threshold", but currently I haven't see a scientific justification for both the 3% and the 95% rule. I do think the cumulative frequency plots can provide more metrics that are also relevant for tag detriment (focusing on the upper range of values), which goes beyond the 3/95% rule. This paragraph already describes the importance of doing this, so I'd encourage the authors to showcase this practice in the current study (as all the ingredients for doing it are there) by liking it more to the visualization in Fig. 3 about the more extreme Gs and/or providing quantitative metrics that are also based on the cumulative frequency plots that describe tag detriment from the upper range of acceleration values (e.g., skewness, weighted area under the curve, etc).

Author's Response to Decision Letter for (RSPB-2021-1207.R0)

See Appendix A.

RSPB-2021-2005.R0

Review form: Reviewer 1

Recommendation
Accept with minor revision (please list in comments)

Scientific importance: Is the manuscript an original and important contribution to its field?
Excellent

General interest: Is the paper of sufficient general interest?
Good

Quality of the paper: Is the overall quality of the paper suitable?
Good

Is the length of the paper justified?
Yes

Should the paper be seen by a specialist statistical reviewer?
No

Do you have any concerns about statistical analyses in this paper? If so, please specify them explicitly in your report.
No

It is a condition of publication that authors make their supporting data, code and materials available - either as supplementary material or hosted in an external repository. Please rate, if applicable, the supporting data on the following criteria.

> **Is it accessible?**
> Yes
>
> **Is it clear?**
> Yes
>
> **Is it adequate?**
> N/A

Do you have any ethical concerns with this paper?
No

Comments to the Author
The authors revisions have greatly improved this manuscript. In particular, the authors have effectively addressed most of my comments from the previous version.

I have a few minor comments regarding this revised manuscript:

Abstract
Lines 55 – 56: It would be useful for the authors to also provide the range of body masses of these four carnivore species here.

Line 56: It's not clear why the term "mostly" is needed here. I suggest removing it.

Line 59: I believe "of" is missing here, "irrespective of the force and time limits specified."

Introduction
Line 69: It should also be noted here that such devices should not influence animal survival in addition to behavior, which would better lead into the subsequent sentences.

Line 94: "accessed" or "assessed"?

Line 97: Remove second "higher" here.

Results
Lines 270 – 271: Did the authors consider including sex as a covariate in their mixed-effects model analysis? Also, with the exception of African lions, it is unclear which species included data from both sexes or only one sex. This should be clarified in both the main text and the supplementary information.

Line 270: The term "appreciable variation" is vague. Please provide statistical values here.

Lines 276 – 279: These lines would be more suitable in the discussion.

Lines 324 – 327: This raises the question of how much data needs to be collected to reliably evaluate 95% limits? The carnivore data ranged from 3 – 21 days and the non-carnivore data from 7 – 168 days. Was this an adequate duration to evaluate cumulative frequencies? Based on these varying durations can the authors recommend a minimum duration for future researchers? The authors' briefly address this topic in the discussion (lines 450 – 453), but I recommend better using their results to inform future research. Additionally, do the authors' findings imply that researchers need to test for sex-specific profiles? This warrants mention in the discussion.

Discussion
Line 395: A word appears to be missing here "deploy systems that about the 3-5% mass rule".

Line 404: Replace "animal" with "animal's".

Figures
Figure 4: The grey points and grey regression line are difficult to discern. I recommend replacing these with black or another darker color.

Supplementary Information
Line 160: Replace "coral" with "corral".

Review form: Reviewer 3

Recommendation
Accept with minor revision (please list in comments)

Scientific importance: Is the manuscript an original and important contribution to its field?
Excellent

General interest: Is the paper of sufficient general interest?
Excellent

Quality of the paper: Is the overall quality of the paper suitable?
Excellent

Is the length of the paper justified?
Yes

Should the paper be seen by a specialist statistical reviewer?
No

Do you have any concerns about statistical analyses in this paper? If so, please specify them explicitly in your report.
Yes

It is a condition of publication that authors make their supporting data, code and materials available - either as supplementary material or hosted in an external repository. Please rate, if applicable, the supporting data on the following criteria.

Is it accessible?
Yes

Is it clear?
Yes

Is it adequate?
No

Do you have any ethical concerns with this paper?
Yes

Comments to the Author
As with the initial submission, I very much applaud the aim and execution of this paper. I think it is a very important contribution to both wildlife biology as a scientific discipline, as well as conservation work.

The authors addressed my main concern with the initial submission (my doubts about the calculated metrics and chosen thresholds) well. Although they kept using their original method, they addressed my concerns well in both the response letter and the manuscript. The manuscript now gives a more nuanced message to the reader, in which it becomes clear that one should not always simply adhere to the 3/95% rule, but that for certain species it is also needed to focus more on peak accelerations and/or higher thresholds. The authors made their arguments clear about why they chose this specific method and message in the manuscript, and I agree with them.

I only have six minor comments:

- I didn't read a clarification about why this study doesn't supply an ethical approval for the lion experiments. Not in the main document, not in the response to reviewers/editor and not in supplemental materials. The associate editor did list this as a concern in the decision letter.
- The supplied data and scripts via figshare are still the same as the initial version. So although the authors replied in their response letter to the main editor that they did attend to this issue, I couldn't find the updated version of the data and scripts.
- L53: It is theoretically incorrect to compare force directly with mass, by stating that a force should be a certain percentage of a mass. Given that force and mass are different physical properties with different units of measurement. I suggest changing the sentence to something like "force which is less than 3% of the gravitational force exerted on the animal's body". I flag this issue here solely in the abstract, but it appears on multiple locations in the manuscript. However, it is most troublesome in the abstract as it hasn't been explained until this point what is actually meant with the comparison between force and body mass.

- L210: Please remove "Studio" behind "R", the version number relates to the R software alone and it is practically irrelevant whether R was used in conjunction with R Studio or not.
- L214: In the response to reviewers the use of a fixed factor is defended for 'collar mass', but in the manuscript it is changed to a covariate nonetheless. Which one is it?
- Fig. 4: Shouldn't the peak acceleration at 0 speed be equal to 1 instead of 0, given that gravity is pulling constantly in one direction without the animal's movement counteracting this? Given that the authors used vectorial sum instead of OBDA, standing still results in exactly 1g force. So the intercept should then be fixed at 1 instead of 0.

Decision letter (RSPB-2021-2005.R0)

28-Sep-2021

Dear Dr Wilson

I am pleased to inform you that your manuscript RSPB-2021-2005 entitled "Animal lifestyle changes acceptable mass limits for attached tags" has been accepted for publication in Proceedings B. This acceptance is provisional-- the persistent issue with ethical approval for lion studies must be fully resolved. There is also a concern about circular arguments that must satisfy the Associate Editor in revision.

The referee(s) have recommended publication, but also suggest some minor revisions to your manuscript. Therefore, I invite you to respond to the referee(s)' comments and revise your manuscript. Because the schedule for publication is very tight, it is a condition of publication that you submit the revised version of your manuscript within 7 days. If you do not think you will be able to meet this date please let us know.

To revise your manuscript, log into https://mc.manuscriptcentral.com/prsb and enter your Author Centre, where you will find your manuscript title listed under "Manuscripts with Decisions." Under "Actions," click on "Create a Revision." Your manuscript number has been appended to denote a revision. You will be unable to make your revisions on the originally submitted version of the manuscript. Instead, revise your manuscript and upload a new version through your Author Centre.

When submitting your revised manuscript, you will be able to respond to the comments made by the referee(s) and upload a file "Response to Referees". You can use this to document any changes you make to the original manuscript. We require a copy of the manuscript with revisions made since the previous version marked as 'tracked changes' to be included in the 'response to referees' document.

Before uploading your revised files please make sure that you have:

1) A text file of the manuscript (doc, txt, rtf or tex), including the references, tables (including captions) and figure captions. Please remove any tracked changes from the text before submission. PDF files are not an accepted format for the "Main Document".

2) A separate electronic file of each figure (tiff, EPS or print-quality PDF preferred). The format should be produced directly from original creation package, or original software format. PowerPoint files are not accepted.

3) Electronic supplementary material: this should be contained in a separate file and where possible, all ESM should be combined into a single file. All supplementary materials accompanying an accepted article will be treated as in their final form. They will be published

alongside the paper on the journal website and posted on the online figshare repository. Files on figshare will be made available approximately one week before the accompanying article so that the supplementary material can be attributed a unique DOI.

Online supplementary material will also carry the title and description provided during submission, so please ensure these are accurate and informative. Note that the Royal Society will not edit or typeset supplementary material and it will be hosted as provided. Please ensure that the supplementary material includes the paper details (authors, title, journal name, article DOI). Your article DOI will be 10.1098/rspb.[paper ID in form xxxx.xxxx e.g. 10.1098/rspb.2016.0049].

4) A media summary: a short non-technical summary (up to 100 words) of the key findings/importance of your manuscript.

5) Data accessibility section and data citation
It is a condition of publication that data supporting your paper are made available either in the electronic supplementary material or through an appropriate repository (https://royalsociety.org/journals/authors/author-guidelines/#data).

In order to ensure effective and robust dissemination and appropriate credit to authors the dataset(s) used should be fully cited. To ensure archived data are available to readers, authors should include a 'data accessibility' section immediately after the acknowledgements section. This should list the database and accession number for all data from the article that has been made publicly available, for instance:
• DNA sequences: Genbank accessions F234391-F234402
• Phylogenetic data: TreeBASE accession number S9123
• Final DNA sequence assembly uploaded as online supplemental material
• Climate data and MaxEnt input files: Dryad doi:10.5521/dryad.12311
NB. From April 1 2013, peer reviewed articles based on research funded wholly or partly by RCUK must include, if applicable, a statement on how the underlying research materials – such as data, samples or models – can be accessed. This statement should be included in the data accessibility section.

If you wish to submit your data to Dryad (http://datadryad.org/) and have not already done so you can submit your data via this link http://datadryad.org/submit?journalID=RSPB&manu=(Document not available) which will take you to your unique entry in the Dryad repository. If you have already submitted your data to dryad you can make any necessary revisions to your dataset by following the above link. Please see https://royalsociety.org/journals/ethics-policies/data-sharing-mining/ for more details.

6) For more information on our Licence to Publish, Open Access, Cover images and Media summaries, please visit https://royalsociety.org/journals/authors/author-guidelines/.

Once again, thank you for submitting your manuscript to Proceedings B and I look forward to receiving your revision. If you have any questions at all, please do not hesitate to get in touch.

Sincerely,
Dr John Hutchinson
mailto: proceedingsb@royalsociety.org

Associate Editor
Board Member
Comments to Author:
Thank you considering all the feedback on the initial submission and for revising your manuscript. Two reviewers have assessed the resubmission, and both are extremely positive but do raise several (valid) minor concerns that would need to be addressed. I am also positive about

the resubmission, but I do feel two additional points need to be highlighted. First the authors did not respond to my query about lion ethics. Further clarification of this is required, I think. It may be solved by a simple explanation, but now all the other experiments are explicitly stated as being covered by ethical approval bodies, while the lion work is not. Second, the now absent third reviewer raised an issue about circulatory in the initial submission: “There is potential to me that the whole framework of the paper is a circular argument. If the accelerations are being taken off the tags when on animals, how do you know that the devices weren’t already impacting acceleration? I.e., your baselines are not realistic baselines.” The authors have attempted to address this at the beginning of the discussion session, but I almost missed it. In other words, I think this language/style here could be a little more direct and little less vague about the issue (without significantly weakening the paper).

Reviewer(s)' Comments to Author:
Referee: 3
Comments to the Author(s).
As with the initial submission, I very much applaud the aim and execution of this paper. I think it is a very important contribution to both wildlife biology as a scientific discipline, as well as conservation work.

The authors addressed my main concern with the initial submission (my doubts about the calculated metrics and chosen thresholds) well. Although they kept using their original method, they addressed my concerns well in both the response letter and the manuscript. The manuscript now gives a more nuanced message to the reader, in which it becomes clear that one should not always simply adhere to the 3/95% rule, but that for certain species it is also needed to focus more on peak accelerations and/or higher thresholds. The authors made their arguments clear about why they chose this specific method and message in the manuscript, and I agree with them.

I only have six minor comments:

- I didn't read a clarification about why this study doesn't supply an ethical approval for the lion experiments. Not in the main document, not in the response to reviewers/editor and not in supplemental materials. The associate editor did list this as a concern in the decision letter.
- The supplied data and scripts via figshare are still the same as the initial version. So although the authors replied in their response letter to the main editor that they did attend to this issue, I couldn't find the updated version of the data and scripts.
- L53: It is theoretically incorrect to compare force directly with mass, by stating that a force should be a certain percentage of a mass. Given that force and mass are different physical properties with different units of measurement. I suggest changing the sentence to something like "force which is less than 3% of the gravitational force exerted on the animal's body". I flag this issue here solely in the abstract, but it appears on multiple locations in the manuscript. However, it is most troublesome in the abstract as it hasn't been explained until this point what is actually meant with the comparison between force and body mass.
- L210: Please remove "Studio" behind "R", the version number relates to the R software alone and it is practically irrelevant whether R was used in conjunction with R Studio or not.
- L214: In the response to reviewers the use of a fixed factor is defended for 'collar mass', but in the manuscript it is changed to a covariate nonetheless. Which one is it?
- Fig. 4: Shouldn't the peak acceleration at 0 speed be equal to 1 instead of 0, given that gravity is pulling constantly in one direction without the animal's movement counteracting this? Given that the authors used vectorial sum instead of OBDA, standing still results in exactly 1g force. So the intercept should then be fixed at 1 instead of 0.

Referee: 1
Comments to the Author(s).
The authors revisions have greatly improved this manuscript. In particular, the authors have effectively addressed most of my comments from the previous version.

I have a few minor comments regarding this revised manuscript:

Abstract
Lines 55 – 56: It would be useful for the authors to also provide the range of body masses of these four carnivore species here.

Line 56: It's not clear why the term "mostly" is needed here. I suggest removing it.

Line 59: I believe "of" is missing here, "irrespective of the force and time limits specified."

Introduction
Line 69: It should also be noted here that such devices should not influence animal survival in addition to behavior, which would better lead into the subsequent sentences.

Line 94: "accessed" or "assessed"?

Line 97: Remove second "higher" here.

Results
Lines 270 – 271: Did the authors consider including sex as a covariate in their mixed-effects model analysis? Also, with the exception of African lions, it is unclear which species included data from both sexes or only one sex. This should be clarified in both the main text and the supplementary information.

Line 270: The term "appreciable variation" is vague. Please provide statistical values here.

Lines 276 – 279: These lines would be more suitable in the discussion.

Lines 324 – 327: This raises the question of how much data needs to be collected to reliably evaluate 95% limits? The carnivore data ranged from 3 – 21 days and the non-carnivore data from 7 – 168 days. Was this an adequate duration to evaluate cumulative frequencies? Based on these varying durations can the authors recommend a minimum duration for future researchers? The authors' briefly address this topic in the discussion (lines 450 – 453), but I recommend better using their results to inform future research. Additionally, do the authors' findings imply that researchers need to test for sex-specific profiles? This warrants mention in the discussion.

Discussion
Line 395: A word appears to be missing here "deploy systems that about the 3-5% mass rule".

Line 404: Replace "animal" with "animal's".

Figures
Figure 4: The grey points and grey regression line are difficult to discern. I recommend replacing these with black or another darker color.

Supplementary Information
Line 160: Replace "coral" with "corral".

Author's Response to Decision Letter for (RSPB-2021-2005.R0)

See Appendix B.

Decision letter (RSPB-2021-2005.R1)

05-Oct-2021

Dear Dr Wilson

I am pleased to inform you that your manuscript entitled "Animal lifestyle changes acceptable mass limits for attached tags" has been accepted for publication in Proceedings B.

You can expect to receive a proof of your article from our Production office in due course, please check your spam filter if you do not receive it. PLEASE NOTE: you will be given the exact page length of your paper which may be different from the estimation from Editorial and you may be asked to reduce your paper if it goes over the 10 page limit.

If you are likely to be away from e-mail contact please let us know. Due to rapid publication and an extremely tight schedule, if comments are not received, we may publish the paper as it stands.

If you have any queries regarding the production of your final article or the publication date please contact procb_proofs@royalsociety.org

Your article has been estimated as being 10 pages long. Our Production Office will be able to confirm the exact length at proof stage.

Data Accessibility section
Please remember to make any data sets live prior to publication, and update any links as needed when you receive a proof to check. It is good practice to also add data sets to your reference list.

Open Access
You are invited to opt for Open Access, making your freely available to all as soon as it is ready for publication under a CCBY licence. Our article processing charge for Open Access is £1700. Corresponding authors from member institutions (http://royalsocietypublishing.org/site/librarians/allmembers.xhtml) receive a 25% discount to these charges. For more information please visit http://royalsocietypublishing.org/open-access.

Paper charges
An e-mail request for payment of any related charges will be sent out shortly. The preferred payment method is by credit card; however, other payment options are available.

Electronic supplementary material:
All supplementary materials accompanying an accepted article will be treated as in their final form. They will be published alongside the paper on the journal website and posted on the online figshare repository. Files on figshare will be made available approximately one week before the accompanying article so that the supplementary material can be attributed a unique DOI.

You are allowed to post any version of your manuscript on a personal website, repository or preprint server. However, the work remains under media embargo and you should not discuss it with the press until the date of publication. Please visit https://royalsociety.org/journals/ethics-policies/media-embargo for more information.

Thank you for your fine contribution. On behalf of the Editors of the Proceedings B, we look forward to your continued contributions to the Journal.

Sincerely,
Editor, Proceedings B
mailto: proceedingsb@royalsociety.org

Appendix A

Dear Dr Hutchinson

Many thanks for your careful consideration of our ms RSPB-2021-1207 entitled "Animal lifestyle changes acceptable mass limits for attached tags" and for the opportunity to resubmit this work as a new manuscript. We have incorporated all suggested changes and are now pleased to resubmit the work for your renewed consideration. We refer to the suggested changes in this cover letter below (*replies in italics*) and have uploaded a ms with tracked changes to make your and the referees' jobs easier.

We believe that the work has substantially improved as a result of the attention given to it by yourself and your reviewers.

We look forward to hearing from you.

Yours sincerely

Rory Wilson and Kayleigh Rose (for the authors).

Dear Dr Wilson:

I am writing to inform you that your manuscript RSPB-2021-1207 entitled "Animal lifestyle changes acceptable mass limits for attached tags" has, in its current form, been rejected for publication in Proceedings B.

This action has been taken on the advice of referees, who have recommended that substantial revisions are necessary. With this in mind we would be happy to consider a resubmission, provided the comments of the referees are fully addressed. However please note that this is not a provisional acceptance.

The resubmission will be treated as a new manuscript. However, we will approach the same reviewers if they are available and it is deemed appropriate to do so by the Editor. Please note that resubmissions must be submitted within six months of the date of this email. In exceptional circumstances, extensions may be possible if agreed with the Editorial Office. Manuscripts submitted after this date will be automatically rejected.

Please find below the comments made by the referees, not including confidential reports to the Editor, which I hope you will find useful. If you do choose to resubmit your manuscript, please upload the following:

1) A 'response to referees' document including details of how you have responded to the comments, and the adjustments you have made.

Done.

2) A clean copy of the manuscript and one with 'tracked changes' indicating your 'response to referees' comments document.

Done.

3) Line numbers in your main document.

Done.

4) Data - please see our policies on data sharing to ensure that you are complying (https://royalsociety.org/journals/authors/author-guidelines/#data).

**Note that the data/code were deemed insufficient in review-- Comments were made that:
"It only contains summarized data and scripts that are used for the linear models. The raw data and code is missing from which the peak accelerations and cumulative frequency plots are constructed that are presented in the paper." and "The data releases are clear and well annotated. However, I do not believe the data and analysis code for the full

cross-species comparison are provided. Only the data and analysis code for the 4 species of free-living carnivores and domestic dogs appear to be available. The cumulative frequency curves of the vectoral sum of the acceleration will have the greatest relevance to future researchers wishing to apply these methods. Hence, it would be useful if these data and analysis code are included." All 3 reviewers raised concerns on this topic and it must be fully addressed in revision.

This has now been attended to. Apologies for providing incomplete data previously.

To upload a resubmitted manuscript, log into http://mc.manuscriptcentral.com/prsb and enter your Author Centre, where you will find your manuscript title listed under "Manuscripts with Decisions." Under "Actions," click on "Create a Resubmission." Please be sure to indicate in your cover letter that it is a resubmission, and supply the previous reference number.

Sincerely,

Dr John Hutchinson, Editor
mailto: proceedingsb@royalsociety.org

Associate Editor
Board Member: 1
Comments to Author:
This paper deals with an important topic in animal research and seeks to influence future policy on animal experimentation that could have important impacts on welfare and scientific practice across a range of biological disciplines. It is therefore no surprise that the reviewers are supportive of the aims and the potential significance of such a study. However, the reviewers vary in their assessment of the quality and robustness of how the current study was carried out. Reviewer 1 provides detailed editorial recommendations that will improve the clarity and discussion currently presented in the manuscript.

These have now been addressed.

Reviewer 3 provides editorial suggestions, but also questions whether the specific metrics extracted from the collected data are the most appropriate for the study goals.

This is now discussed in the ms.

Reviewer 2 is more critical and questions more fundamental aspects of the study such as its design and sample size. My feeling is that nearly all the comments made by the reviewers require very careful consideration, which will likely result in some re-analysis and potentially collection of some additional data. For example, it might be possible to address a number of the concerns raised by reviewer 1 through analysis of new/additional data from a small sample of domestic dogs. As a further point, the reviewers note that not all raw data required to repeat the analyses were available with the first submission, and that ethical approval (i.e. by an ethics committee) for the lion study was not stated.

Reviewer(s)' Comments to Author:

Referee: 1

Comments to the Author(s)
This manuscript presents a novel approach for evaluating the force imposed by animal-attached devices. As such, it addresses an important topic regarding the appropriate mass of these devices and should be of broad interest to ecologists and wildlife biologists. In particular, the authors highlight an important point that animal athleticism and behavior should be considered when evaluating tag suitability in addition to body mass. Overall, the manuscript is well written and the analyses are suitable in addressing the research topic.

Thank you for this.

My comments below focus on clarifying details of the manuscript that are unclear in some areas:

Abstract

Lines 57 – 58: These analyses all relate to data from carnivores. That should be clarified here.

Done.

Introduction

Lines 100 – 100: I recommend the authors reference Brander and Cochran (1969) here. This reference provides an approximate 5% justification under the criteria that the weight of the tag does not cause an apparent change in behavior patterns. Additionally, I recommend the authors expand on this statement. There are a wealth of studies that have reviewed whether the 3 – 5% rule is appropriate.

We have now referenced this and added others that have reviewed the 3-5% rule.

Lines 110 – 121: The ordering here is confusing as it is unclear how the non-carnivore data is being used. I recommend either combining the carnivores and non-carnivores in this text or separately explaining how the data from the non-carnivores is used in comparison to the data from the carnivores and domestic dogs.

We have now consolidated the carnivores with the other wild animals and put the domestic dog paragraph at the end.

Materials and Methods

Line 139: It would be useful to know the weights of these collars, mean and range.

Fair point. The collar masses were standardized so there is effectively no range for those but we have now stated that all collars represented <3% of the animal masses and referred the readers to a supplemental table which details the masses of all species collared in the ms as well as information on the mass of their collars (see point below).

Additionally, a Supplemental table listing each species and the collar mass used and its percent mass relative to the species body mass is needed.

Done. See point above.

Lines 146 – 149: Provide the mean mass of each species as was done for the carnivores.

Done.

Lines 146 – 149: This is redundant with the introduction. I recommend shortening this to just a list of the species rather than listing the Family names again.

The family names have now been deleted as suggested.

Line 149: It would again be useful to know the weights of these collars, mean and range.

This detail has now been provided in the supplementary table and, as with the carnivores, we have mentioned that in all cases the devices constituted <3% of the animals' masses. If preferred, we can, of course, add the specifics of each collar mass to the main text but it tends to clutter the flow.

Lines 178 – 181: I believe all species (including the non-carnivores) had raw acceleration converted to the vectorial sum. It would be preferable to indicate that here.

Apologies. We should have been specific and have now done this.

Lines 178 – 181: Typically, dynamic acceleration (ODBA or VeDBA) is used rather than raw acceleration that includes the effects of gravity. It would be helpful for the authors to explain why they have chosen to use raw acceleration rather than dynamic acceleration.

This has now been done.

Results

Lines 225 – 227: The significance of acceleration exceeding 1 g in that it will result in force exceeding 3% (assuming a 3% tag mass) should be explained in the introduction or methods.

This has now been clarified in the introduction.

Lines 225 – 227: I believe this refers to the percentage time during each specific behavior (walk, trot, bound). If so, that should be clarified here and in the table heading to Table S1.

Done.

Line 230: I believe this should refer to Figure 2 not Figure 1.

No, this is actually Fig. 1, as stated. Fig. 1 is complex though, so we have added clarification to indicate where, exactly, the stated condition is fulfilled.

Lines 256 – 257: This sentence is confusing at the start of the paragraph. Suggest rewording.

Reworded.

Lines 259 – 261: Why do the authors assume the tags amounted to 3% of their body mass rather than using the actual mass of the collars that were deployed? As I mention in my comments for lines 354 – 358, a tag (collar) that is 3% of the mass of a 180 kg lion seems unlikely to be deployed.

This has now been changed to accommodate the referee's suggestion.

Figure 3: Again, I question whether it is reasonable to assume a tag that is 3% of the body mass of a lion. It seems unlikely that such a heavy tag (collar) would be deployed regardless of the 3 – 5% justification. It would be informative to evaluate how these results would compare using a typical collar mass for a lion (e.g., ~1.2 kg) as well as the 3% mass the authors present here.

We have now changed Fig. 3 to incorporate this suggestion and show how the peak forces relate to the collars actually worn by the lions (with differences highlighted between females and males as a percentage of their respective body masses).

Figure 5A: The blue arrows and species labels in this figure could be removed. I found them to be confusing particularly since the Marten arrow is pointing at the Koala line.

Removed.

Figure 5B: I suggest labeling the Z axis as "Derived tag mass as percentage of body mass". Currently it is not apparent that this value is derived from the corresponding measure of force.

This has been changed as suggested.

Lines 300 – 303: For future researchers to apply these methods, it would be helpful to describe how the authors determined when animals were active versus inactive.

This has now been done.

Discussion

Lines 346 – 347: Again, the significance of tag forces > 1 g should be explained prior to this.

We have done this though note a slight risk of repetition.

Lines 348 – 351: It would be good to qualify that the 3-5% mass limits seem appropriate for slow-moving animals based on the measure of force, which does not mean such tags may not nevertheless influence these animals' behavior.

Done.

Lines 354 – 358: Though a tag 3% of the mass of a lion seems unlikely (i.e. 5.4 kg). In extremely large animals the 3% rule is less likely to be a limiting factor. Whereas in smaller species it is more likely to influence tag restrictions. It would be worth noting this point in the discussion.

Thank you for this important point. It has now been incorporated in the text.

Line 356: Do the authors' findings suggest that energy expenditure estimates derived from acceleration may be impacted by the weight of the tag? How does the tag driven force influence DBA measurements?

We have now included text that mentions this – albeit further down in the discussion at the point where we discuss tag detriment.

Lines 360 – 379: These are important points. Did the authors consider testing this in their dog trials (comparing the forces of mounted tags versus collar mounted tags)?

Yes, we did, but this entails a whole series of new experiments and would greatly expand the length of the ms. We are conducting work on this aspect for a future publication though.

Lines 404 – 406: It would be helpful to clarify here that the "95% of the time" refers to "95% of the time while active".

Good point. This has now been specified.

Lines 425 – 427: This discussion ignores the issue of battery life and weight, which is often the heaviest component of wildlife tags. Human wearables are able to use rechargeable batteries that may last for hours to days before needing to be recharged. Such recharging options are typically not available or suitable for wildlife applications. I recommend highlighting the need for either more energy efficient sensors and/or advances in battery performance and longevity.

Indeed. This is an important point that we have now made. Thank you.

Supplementary Materials

Lines 25 – 30: The main text indicates data were collected during normal activities in the wild. It's unclear whether this cheetah data was collected in the wild?

This has now been clarified.

Line 54: Please provide details on what type of collars these were.

Done.

Lines 67 – 70: Details on pine martins are provided on lines 15 – 23. I believe these lines can be deleted.

Done.

Line 80: Provide details on what type of collars these were.

Done.

Line 121: Less than 80 g is vague. Can the authors provide a more precise value here or provide a range?

Done.

Data Release

I do not believe the data and analysis code for the full cross-species comparison are provided. The cumulative frequency curves of the vectoral sum of the acceleration will have the greatest relevance to future researchers wishing to apply these methods. Hence, it would be useful if these data and analysis code are included.

Apologies. These data are now included. The analysis that we used for this was DDMT, a program sold by Wildbyte Technologies so not in the public domain. However, cumulative frequency plots are easy to construct in Excel, Origin, R etc.

Referee: 2

Comments to the Author(s)
This is an interesting well-written paper addressing an important issue. The approach to biologging certainly does need this kind of thinking. I do wonder if it would be better suited to Methods in Ecology and Evolution.

Thank you for these positive comments. We did, in fact, submit the work to Methods in Ecol and Evol. but they said that the description of the methods was not long enough for them to consider it. We would have thought that it should be the value or implications of the method that would be most important but then we do not make MEE rules.

Major Comments

1. There is potential to me that the whole framework of the paper is a circular argument. If the accelerations are being taken off the tags when on animals, how do you know that the devices weren't already impacting acceleration? I.e., your baselies are not realistic baselines.

Thank you for this. We have included an extra paragraph at the beginning of the discussion to deal with this interesting point.

2. Species chosen – I think more rationale needs to be added here. At the moment, it reads like a slightly random list of animals, that likely were down to availability and ease of access. That is fine, in a way, but really needs a better argument. The rationale presented is incredibly brief, and doesn't really fully define the life history of the species.

We have now expanded this section to clarify this.

3. Sample sizes – some are incredibly small (N = 1). Should a study really be advocating an entirely new approach to decision making when it comes to biologging (which I fully support) on such small sample sizes?

While it is true that the sample sizes are small, interspecific differences are clear (with the variance around mean cumulative plots – see Fig. 5a – highlighting this). In fact, the cheetah is the only species where we have an N of 1 and we included this because of its well-known spectacular athleticism - all other species had an N of 5 or greater,. We note that this work is intended to provide a framework within which people may work rather than provide the community with species-specific definitive values.

4. Dog gaits – what criteria did you use to visually inspect gaits? This seems somewhat superficial. These need clear definitions, and how many observers decided on these gaits just using their eyes?

The reviewer is correct. The dogs were encouraged by their owners to traverse the tracks at a slow, moderate, and fast speeds. We can therefore justify categorizing into three groups. Given that there are eight canine gaits which are selected differently according to breed, body size and speed, and that we have worked with only 12 domestic dogs, the specific details of the gait are not provided here. We have renamed our three categories as slow (walk/amble) moderate (pace/trot) and fast (Canter/Gallop) and have made this clear in the methods.

5. Gait – in the Introduction and Methods, gait is now a major point, but then in the Results, is given much prominence. This leads, as a reader, to an element of mis-match.

We are not clear what is meant by this but think that the referee means that gait is not given enough prominence in the Introduction and the Methods. As such, we have mentioned gait in the Introduction and added the explanation that gait is a major modulator of acceleration signature to our methods.

Minor Comments

• Latins need adding for certain species.

Apologies for omitting these. This is now done.

• Line 78: I think smart tag is a specific term? Either needs to be defined or just use biologger.

We have defined the term rather than use the term 'biologgers' since transmission (rather than logging) tags have also been used in this context.

• Line 85: Can you be more specific about what the effect on the penguin populations was? A reduction one assumes? 1%? 85%

This has now been clarified.

• Line 157: Is there an issue with the owners providing body mass of their dogs? Potential for bias? Heavily inaccurate scales?

We ensured that owners provided the most recent body mass recorded by their vets. 4/12 dogs were provided by the RSPCA and were weighed carefully. We ascertained by using the other biometrics that we took of the dogs that the values given were likely to be appropriate.

• Line 220: "most likely corresponded" - I think this needs to be somewhat more stringent?

In an attempt to save space, we perhaps underplayed our hand. We have now clarified our process more thoroughly.

• Discussion – a random opening, given the theme of the paper. A useful point to make, but feel the Discussion would work better opening with some confirmation of the primary results.

Agreed. We now open the discussion with a topic suggested by referee 1.

• Some minor referencing issues.
We have now re-checked the references.

Referee: 3

Comments to the Author(s)
Main comments:
I applaud the aim and execution of this paper to scrutinize the (largely unsubstantiated) rule-of-thumb used for animal-born tag mass. I think the presented cumulative frequency graphs and the proposed method for determining maximum tag masses based on g-forces exerted on animals are interpretable and appropriate, which could lead to a quick change in guidelines and practices used by animal researchers and ethical committees.

Thank you for these encouraging words.

However, my main concern with the presented method is the focus on the 95% lowest accelerations as a limit for tag mass, where (as the authors already mention themselves in L396) it are often the periods with the most extreme g-forces that impact animal the most (either through direct strain of the forces exerted on the body or through indirect effects by e.g. impacting hunting success). Shifting the focus of the method to the upper range of the g-forces would therefore do more justice to these considerations, for which I make suggestions below.

This is a fair point that caused us some concern ourselves. We welcome this suggestion and have modified the text accordingly.

Specific comments:
L2: "changes" may at first be read as a noun instead of a verb. Perhaps "affects" or "influences" is better.

Modified as suggested.

L79: "scales" is multi-interpretable in this context.

We have now specified these scales.

L101: This undermines the recommendation in the abstract of targeting <3% body weight in force for 95% of the time. Why should we keep adhering to this 3% rule?

We have amplified on this following suggestions by another referee.

L138: Please also provide the sensitivity or resolution of the accelerometer, so that we can get an impression of the granularity of the data (as 0-32g is quite a large range, the data could probably be quite granular, which may impact the accurate determination of the force belonging to 3% body weight).

It is fortunate that we revisited this following the comment. After consultation with the tag manufacturers, it transpires that the measurement range was only up to 16 g (which, however, easily covered the maximum g values that occurred in our work). This gave us a resolution of 0.00049 g. This has now been stated in the text.

L140: For me it is not clear if all components attached to the collar were in the same spot as the accelerometer. Is there for example not a counterweight on the other side of the collar to prevent rotation? Or the battery at the other end of the collar compared to the sensor case? This setup is critical to know for the conversion to forces on the animal body.

This has now been clarified. The loggers were placed on the collars with the batteries (and sometimes extra weighting – see e.g. the dog trials - to keep the measuring system and weighting ventral and comparable between species). However, since the collars could rotate, there were times when the package rotated off the ventral position for a short period.

L144-149: This is almost a copy of the paragraph in the introduction, I would remove one of them.

Done.

L149: I would prefer to know the weight distribution of the collars of these animals in the main text as well (just as for the carnivores and the dogs), as this is important information for the reader as to how they should compare the results for these different groups.

This has now been clarified – see above.

L162: So the load was spread equally along the collar ("along their full-length")? Please make this 100% clear in the text, because to me this contradicts with "stacked" (perhaps because I'm not a native speaker, but many people aren't). If it's equally spread, then it does however differ from the situation with the carnivores where the load was positioned (presumably?) at one location on the collar.

This was due to the greatest collar masses for the big dogs requiring large amounts of lead, which came in the form of plates, up to 10 cm long. These were bent to replicate the collar circumference and were stacked upon each other before being attached with tape to the collar. We have clarified this in the text.

L164: Given that it's the same accelerometer, it is not needed to give all specs again.

Again, fair enough. We have deleted the specifications.

L170: Not "gallop" or "canter"? "Bound" is used almost everywhere in the text, but once "gallop" is used as well. I would check which name is used more often in the literature for this gait.

Sorry for having used more than one term. Gallop has been removed and replaced by bound (which is the appropriate generic term for that gait across taxa).

L187: But m is the mass of the entire collar, not of the sensor, right? If m is distributed equally along a collar then the acceleration of the sensor does not apply to the whole mass in the same way.

Yes, quite right (we should have specified and have done now). In fact, the vast majority of the mass was withheld within the tag (collars should be as light as possible) so, although

L198: Please give R version instead of RStudio version. Also lme4 version, as those two versions primarily determine the functioning of the code.

Done

L201: Why isn't collar mass a covariate? Even though there are only a few categories, there certainly is an order in these (0<1<2<3).

This is true. However, the tag % body mass has been included as a factor here in order to to generate four separate slopes (or lack of slopes) for figure 4 and the supplementary figures.

L209: Reported where?

This has now been clarified in the legend for supplementary table S6 instead.

L218-220: Determined how?

Clarified.

L221-222: What is meant with the text about dogs here?

We have rewritten this to make it clearer.

L223: So for clarification: the cumulative frequency plots did not use the peak accelerations?

That is exactly right. We did not use peak accelerations for these cumulative frequency plots.

To quantify strain on the animal, I would be inclined to make frequency plots solely of these peak acceleration values instead of all acceleration values. This has the benefit that it focuses on the maximum force of each cyclic period, thereby giving more attention in the method to the highest forces exerted by the tag (which ultimately determines the effect on the animal the most, not the 95% lowest forces). Furthermore, based on our own experiences we can state that for each step that we take (which these acceleration periods mostly are) the forces that impact us the most are the maximum values, e.g., when our feet hit the ground during running. Or compared to horse riding: it are the peak downward forces of the rider on the horse's back during trotting that determine the strain of the saddle on the horse. So why take all the lower forces also into account in the frequency plots when these do not really matter for the animal? Furthermore, it makes the analysis more coherent when it is centred all around these peak accelerations, because currently the analyses considering the peak acceleration in carnivores and dogs are somewhat disjunct from the cumulative frequency plots.

The method suggested has real value, which is why we present such data for the carnivores – which highlights exactly the issue pointed out by the referee. However, adopting that method across all data presents a number of problems and biases. Firstly, it assumes that all acceleration signals have a clean wave function, which many animal behaviours do not. Secondly, even in clean waveforms of some animals – such as an albatross exposed to high centripetal acceleration during dynamic soaring [note that although we streamline our ms to collar-wearing animals, the approach is valid for any animal wearing a tag]– the length of time representing the build-up to the peak can be highly variable. For example, the bird can have a 10 s period during which time there is a single peak in the vectorial sum of the acceleration of e.g. 2.6 g. However, within the cycle, the bird can be exposed variable periods (e.g. 2 to 5 s) where the vector sum is >2 g. Obviously, this means that there is a substantial difference of the length of time that the bird is subject to higher tag forces, with assumed related consequences. In other words, although the peak accelerations are the time where the greatest instantaneous detriment is expected, the integral of all the forces over time represents the true situation. This can only be achieved if all data are considered.

L263: What does the x-axis mean? Shouldn't it be "time since start of chase"?

We used a percentage of time into the chase for the x-axis because the data are derived from chases of variable length. We have slightly modified the figure caption to make this clearer.

L268: Why do the trend lines continue before 0% and after 100%?

Yes. We appreciate that this is confusing. It was originally done to show the relationship to the baseline (1 g) but we realise that this did not help. We have now redone the graph and represented it in two plots to highlight the difference between male and female lions, which had different mass collars which therefore produced difference tag forces exerted as a percentage of body mass.

L276: Why are there two models specified in Table S4 (A and D) for peak acceleration? A also has non-significant variables included.

The different statistical tests are included to explain what is being presented different graphs – one is for the relationship with speed, another with period, in a similar manner to the analysis with the four wild carnivores. Only non-significant interaction terms were removed from the models. Whether or not there are independent effects of different parameters needs to be demonstrated statistically here, so mass was not removed from the model.

L280: But the this moving object did not have this 1-3% mass, right? So even though the movement was exacerbated, the force exerted on the animal's body presumably not (as it rotated along the neck, instead of pounding in one place).

This sentence refers to the 3% scenario with the variability in forces exerted increasing (now modified to make this clearer). It is specifically the value of the vector sum of the forces (derived from the acceleration vector sum and the mass of the tag) that quantifies the forces exerted on the animal's body so the figure cited refers to that force and its variation. We do not discuss the direction of the force applied in this ms (given by the vector direction) but it will vary if/when the collar rotates.

L284-286: Why isn't the intercept fixed? With a speed of 0, the vectorial sum g-force is 1 (gravity), so you can fix the intercept at 1. This reduced the degrees of freedom of the model fit and allows for a more straight-forward comparison of the slopes of the different tags.

The intercept has now been fixed. Thank you for pointing this out.

L399-402: I fully agree, but I posit this paper can already contribute more to this problem by acknowledging that the peak accelerations matter the most. Or alternatively in some other way provide a measure that focuses more on the highest g-forces exerted on the animal. In the currently proposed 3/95%-threshold method it does not matter how high the g-forces become after the 95% threshold, but the method of constructing the cumulative frequency graph does allow for it. Only focussing on the amount of time where the tag exerts more than 3% body weight in force reduces the dimensionality of the strain quite a lot.

This highlights the problem of scarce, but substantial, forces which may influence detriment disproportionately. To incorporate this point, we have now added text that points out how peak forces per stride may be particularly critical in some behaviours. This should alert readers to the problem and also refers to our peak acceleration data.

L413: Most readers will solely read the abstract and act according to that, so they will assume the authors do advocate for this 3/95% rule. Stating here that the authors do not advocate the 3% rule does not seem to match with the importance of the 3% rule in the method and the prominent place it has in the abstract. In the abstract it is even stated that 3/95% is a "scientifically robust acceptable threshold", but currently I haven't see a scientific justification for both the 3% and the 95% rule. I do think the cumulative frequency plots can provide more metrics that are also relevant for tag detriment (focusing on the upper range of values), which goes beyond the 3/95% rule. This paragraph already describes the importance of doing this, so I'd encourage the authors to showcase this practice in the current study (as all the ingredients for doing it are there) by liking it more to the visualization in Fig. 3 about the more extreme Gs and/or providing quantitative metrics that are also based on the cumulative frequency plots that describe tag detriment from the upper range of acceleration values (e.g., skewness, weighted area under the curve, etc).

This comment highlights the difficulties of trying to change long-term practice – in this case the 3-5% rule - and the referee believes that we should not act as if 3% is the right answer, and nor is using the 95% cumulative limits. We agree. However, somehow, we have to wean people off preconceived ideas with least complexity otherwise they will not consider a change. We have taken the comments on board though, and changed the abstract to say that the 3%, or some other, limit may be used and that people should use a minimum of the 95% limits on the cumulative acceleration plot (or higher). The main text also makes this clear. We think that this allows people to reflect on the 3% rule and where, exactly the cumulative distribution cut-off point may occur.

On the note of looking more into the cumulative frequency plots at the higher acceleration/force values, we would be happy to add more figures (which showed, for example, what happens at the top end) but we already have five figures and to expand this further seems excessive given the instructions to authors. Perhaps the editor could advise on this?

****

Journal Name: Proceedings of the Royal Society B
Journal Code: RSPB

Print ISSN: 0962-8452
Online ISSN: 1471-2954
Journal Admin Email: proceedingsb@royalsociety.org
MS Reference Number: RSPB-2021-1207
Article Status: REJECTED
MS Dryad ID: RSPB-2021-1207
MS Title: Animal lifestyle changes acceptable mass limits for attached tags
MS Authors: Wilson, Rory; Rose, Kayleigh; Gunner, Richard; Holton, Mark; Marks, Nikki; Bennett, Nigel; Bell, Stephen; Twining, Joshua; Hesketh, Jamie; Duarte, Carlos; Bezodis, Neil; Jezek, Milos; Painter, Michael; Silovsky, Vaclav; Harel, Roi; Crofoot, Meg; Arnould, John; Allan, Blake; Whisson, Desley; Alagaili, Abdulaziz; Scantlebury, David
Contact Author: Rory Wilson
Contact Author Email: r.p.wilson@swansea.ac.uk
Contact Author Address 1: Swansea University
Contact Author Address 2: Singleton Park
Contact Author Address 3:
Contact Author City: Swansea
Contact Author State:
Contact Author Country: United Kingdom of Great Britain and Northern Ireland
Contact Author ZIP/Postal Code: SA2 8PP
Keywords: Detriment, Welfare, Ethics, Tag
Abstract: Animal-attached devices have transformed our understanding of vertebrate ecology. To minimize tag-related harm, researchers have long advocated that tag masses should not exceed 3% of the animal's body mass. However, this ignores tag forces generated by animal movement. Using data from collar-attached accelerometers on diverse free-ranging terrestrial animals, we detail a tag-based acceleration method in which we quantify animal athleticism in terms of fractions of animal movement time devoted to different collar-recorded accelerations. The varying accelerations are converted to forces imposed on the animals based on the tag mass and allow derivation of defined force limits, for specified fractions of any animal's active time. We demonstrate how species athleticism is the principal determinant of tag forces, whereas body mass is of little importance. Forces exerted by '3%' tags were mostly equivalent to 4-19% of the animals' masses during moving, with a maximum of 54% in a hunting cheetah. Specifying that tags should exert forces that are less than 3% of the animal's body mass for 95% of the time led to corrected tag masses constituting between 1.6% and 2.98 body mass, with values depending on animal athleticism. Our findings change how acceptable tag mass limits should be determined by ethics bodies.
EndDryadContent

Appendix B

Dear Dr Hutchinson

Many thanks for your encouraging news that our manuscript has been accepted for publication in Proceedings B subject to minor changes. We have instigated all the suggested changes and refer to them explicitly below (our comments in italics).

Many thanks for your careful attention.

Sincerely

Rory Wilson and Kayleigh Rose (for the authors).

I am pleased to inform you that your manuscript RSPB-2021-2005 entitled "Animal lifestyle changes acceptable mass limits for attached tags" has been accepted for publication in Proceedings B. This acceptance is provisional-- the persistent issue with ethical approval for lion studies must be fully resolved. There is also a concern about circular arguments that must satisfy the Associate Editor in revision.

This has been attended to. See below.

The referee(s) have recommended publication, but also suggest some minor revisions to your manuscript. Therefore, I invite you to respond to the referee(s)' comments and revise your manuscript. Because the schedule for publication is very tight, it is a condition of publication that you submit the revised version of your manuscript within 7 days. If you do not think you will be able to meet this date please let us know.

To revise your manuscript, log into https://mc.manuscriptcentral.com/prsb and enter your Author Centre, where you will find your manuscript title listed under "Manuscripts with Decisions." Under "Actions," click on "Create a Revision." Your manuscript number has been appended to denote a revision. You will be unable to make your revisions on the originally submitted version of the manuscript. Instead, revise your manuscript and upload a new version through your Author Centre.

When submitting your revised manuscript, you will be able to respond to the comments made by the referee(s) and upload a file "Response to Referees". You can use this to document any changes you make to the original manuscript. We require a copy of the manuscript with revisions made since the previous version marked as ‘tracked changes’ to be included in the ‘response to referees’ document.

All this attended to as requested, the tracked changes for the main manuscript and supplementary materials are below our response in this document.

Before uploading your revised files please make sure that you have:

1) A text file of the manuscript (doc, txt, rtf or tex), including the references, tables (including captions) and figure captions. Please remove any tracked changes from the text before submission. PDF files are not an accepted format for the "Main Document".

Done.

2) A separate electronic file of each figure (tiff, EPS or print-quality PDF preferred). The format should be produced directly from original creation package, or original software format. PowerPoint files are not accepted.

Done.

3) Electronic supplementary material: this should be contained in a separate file and where possible, all ESM should be combined into a single file.

Done.

All supplementary materials accompanying an accepted article will be treated as in their final form. They will be published alongside the paper on the journal website and posted on the online figshare repository. Files on figshare will be made available approximately one week before the accompanying article so that the supplementary material can be attributed a unique DOI.

Online supplementary material will also carry the title and description provided during submission, so please ensure these are accurate and informative.

Done.

Note that the Royal Society will not edit or typeset supplementary material and it will be hosted as provided. Please ensure that the supplementary material includes the paper details (authors, title, journal name, article DOI). Your article DOI will be 10.1098/rspb.[paper ID in form xxxx.xxxx e.g. 10.1098/rspb.2016.0049].

Done.

4) A media summary: a short non-technical summary (up to 100 words) of the key findings/importance of your manuscript.

Done, and added to the main text.

5) Data accessibility section and data citation
It is a condition of publication that data supporting your paper are made available either in the electronic supplementary material or through an appropriate repository (https://royalsociety.org/journals/authors/author-guidelines/#data).

In order to ensure effective and robust dissemination and appropriate credit to authors the dataset(s) used should be fully cited. To ensure archived data are available to readers, authors

should include a 'data accessibility' section immediately after the acknowledgements section. This should list the database and accession number for all data from the article that has been made publicly available, for instance:

- DNA sequences: Genbank accessions F234391-F234402
- Phylogenetic data: TreeBASE accession number S9123
- Final DNA sequence assembly uploaded as online supplemental material
- Climate data and MaxEnt input files: Dryad doi:10.5521/dryad.12311

NB. From April 1 2013, peer reviewed articles based on research funded wholly or partly by RCUK must include, if applicable, a statement on how the underlying research materials – such as data, samples or models – can be accessed. This statement should be included in the data accessibility section.

If you wish to submit your data to Dryad (http://datadryad.org/) and have not already done so you can submit your data via this link http://datadryad.org/submit?journalID=RSPB&manu=(Document not available) which will take you to your unique entry in the Dryad repository. If you have already submitted your data to dryad you can make any necessary revisions to your dataset by following the above link. Please see https://royalsociety.org/journals/ethics-policies/data-sharing-mining/ for more details.

We have now done this, and provided the appropriate reference.

6) For more information on our Licence to Publish, Open Access, Cover images and Media summaries, please visit https://royalsociety.org/journals/authors/author-guidelines/.

Once again, thank you for submitting your manuscript to Proceedings B and I look forward to receiving your revision. If you have any questions at all, please do not hesitate to get in touch.

Sincerely,

Dr John Hutchinson
mailto: proceedingsb@royalsociety.org

Associate Editor
Board Member
Comments to Author:
Thank you considering all the feedback on the initial submission and for revising your manuscript. Two reviewers have assessed the resubmission, and both are extremely positive but do raise several (valid) minor concerns that would need to be addressed. I am also positive about the resubmission, but I do feel two additional points need to be highlighted. First the authors did not respond to my query about lion ethics. Further clarification of this is required, I think. It may be solved by a simple explanation, but now all the other experiments are explicitly stated as being covered by ethical approval bodies, while the lion work is not.

Apologies for this. We are not sure how we forgot the information in our last iteration. It has now been added to the supplementary information. Specifically, we say; ***Conditions, permits and approvals for fieldwork on lions were granted from South African National Parks (Permit***

Number SCAM 1550), from Swansea University College of Science Ethics Committee (SU) (SU-Ethics-Student-260919/1229), the Animal Welfare Ethical Review Committee at Queens University Belfast (QUB-BS-AREC-18-006) and the University of Pretoria (NAS061-19).

Second, the now absent third reviewer raised an issue about circulatory in the initial submission: “There is potential to me that the whole framework of the paper is a circular argument. If the accelerations are being taken off the tags when on animals, how do you know that the devices weren’t already impacting acceleration? I.e., your baselines are not realistic baselines.” The authors have attempted to address this at the beginning of the discussion session, but I almost missed it. In other words, I think this language/style here could be a little more direct and little less vague about the issue (without significantly weakening the paper).

Of course. We have now changed the language style to make the issue more obvious.

Reviewer(s)' Comments to Author:

Referee: 3

Comments to the Author(s).
As with the initial submission, I very much applaud the aim and execution of this paper. I think it is a very important contribution to both wildlife biology as a scientific discipline, as well as conservation work.

Thank you for these kind words.

The authors addressed my main concern with the initial submission (my doubts about the calculated metrics and chosen thresholds) well. Although they kept using their original method, they addressed my concerns well in both the response letter and the manuscript. The manuscript now gives a more nuanced message to the reader, in which it becomes clear that one should not always simply adhere to the 3/95% rule, but that for certain species it is also needed to focus more on peak accelerations and/or higher thresholds. The authors made their arguments clear about why they chose this specific method and message in the manuscript, and I agree with them.

We are very happy to hear this.

I only have six minor comments:

- I didn't read a clarification about why this study doesn't supply an ethical approval for the lion experiments. Not in the main document, not in the response to reviewers/editor and not in supplemental materials. The associate editor did list this as a concern in the decision letter.

Again, apologies for this. The omission is inexcusable, especially since it was flagged up in two places. We have added the following information to the SI; ***Conditions, permits and approvals for fieldwork on lions were granted from South African National Parks (Permit Number SCAM 1550), from Swansea University College of Science Ethics Committee (SU) (SU-Ethics-***

Student-260919/1229), the Animal Welfare Ethical Review Committee at Queens University Belfast (QUB-BS-AREC-18-006) and the University of Pretoria (NAS061-19).

- The supplied data and scripts via figshare are still the same as the initial version. So although the authors replied in their response letter to the main editor that they did attend to this issue, I couldn't find the updated version of the data and scripts.

A big apology from KR. I followed the link to dryad in an email from ProcRSocB and assumed this meant you had a direct link also. Here is the temporary link from dryad while the resources are 'private for peer review'.

https://datadryad.org/stash/share/52PkFJr_RTi-s9_xWgl3O_Z9up2scWiSjFwhjT5sKM4.

And the doi

https://doi.org/10.5061/dryad.rjdfn2zbm

We have updated the data availability statement.

- L53: It is theoretically incorrect to compare force directly with mass, by stating that a force should be a certain percentage of a mass. Given that force and mass are different physical properties with different units of measurement. I suggest changing the sentence to something like "force which is less than 3% of the gravitational force exerted on the animal's body". I flag this issue here solely in the abstract, but it appears on multiple locations in the manuscript. However, it is most troublesome in the abstract as it hasn't been explained until this point what is actually meant with the comparison between force and body mass.

This is a good point and we were perhaps remiss in trying to simplify the text to make it conceptually more accessible to readers. We have now changed the text throughout to remedy this.

- L210: Please remove "Studio" behind "R", the version number relates to the R software alone and it is practically irrelevant whether R was used in conjunction with R Studio or not.

Done.

- L214: In the response to reviewers the use of a fixed factor is defended for 'collar mass', but in the manuscript it is changed to a covariate nonetheless. Which one is it?

Clarified.

- Fig. 4: Shouldn't the peak acceleration at 0 speed be equal to 1 instead of 0, given that gravity is pulling constantly in one direction without the animal's movement counteracting this? Given that the authors used vectorial sum instead of OBDA, standing still results in exactly 1g force. So the intercept should then be fixed at 1 instead of 0.

Yes. Apologies. This has been corrected.

Referee: 1

Comments to the Author(s).
The authors revisions have greatly improved this manuscript. In particular, the authors have effectively addressed most of my comments from the previous version.

Thank you for this.

I have a few minor comments regarding this revised manuscript:

Abstract
Lines 55 – 56: It would be useful for the authors to also provide the range of body masses of these four carnivore species here.

Done.

Line 56: It's not clear why the term "mostly" is needed here. I suggest removing it.

Done.

Line 59: I believe "of" is missing here, "irrespective of the force and time limits specified."

Apologies. Now corrected.

Introduction
Line 69: It should also be noted here that such devices should not influence animal survival in addition to behavior, which would better lead into the subsequent sentences.

Changed.

Line 94: "accessed" or "assessed"?

Changed.

Line 97: Remove second "higher" here.

Removed.

Results
Lines 270 – 271: Did the authors consider including sex as a covariate in their mixed-effects model analysis? Also, with the exception of African lions, it is unclear which species included data from both sexes or only one sex. This should be clarified in both the main text and the supplementary information.

Thank you for pointing this out. This has now been clarified in the main text and in Table S1. We did not include sex as a covariate in the mixed-effects models as, for some species, there were only data from individuals of one sex or there was a heavily biased sex ratio, e.g. 9 females and 3 male domestic dogs. We do, however, bring attention to the potential for sex differences e.g. the example in hunting lions, and draw attention to this in the discussion.

Line 270: The term “appreciable variation” is vague. Please provide statistical values here.

This has been clarified by providing mean values and referencing Fig. 3, which shows the means for female and male lions as well as IQRs for both combined. If this is to be further expanded, we could split the IQRs for the females and males separately although, trying this, we noted that the graph was much more cluttered and it is difficult to allocate the whiskers to lion sex.

Lines 276 – 279: These lines would be more suitable in the discussion.

Now moved to the discussion.

Lines 324 – 327: This raises the question of how much data needs to be collected to reliably evaluate 95% limits? The carnivore data ranged from 3 – 21 days and the non-carnivore data from 7 – 168 days. Was this an adequate duration to evaluate cumulative frequencies? Based on these varying durations can the authors recommend a minimum duration for future researchers? The authors’ briefly address this topic in the discussion (lines 450 – 453), but I recommend better using their results to inform future research. Additionally, do the authors’ findings imply that researchers need to test for sex-specific profiles? This warrants mention in the discussion.

This is an important point but one that would require an extensive new section if we were to consider how the cumulative frequency curves of the vectorial sum of the acceleration changes over time for our species. In order to make this critical point, we have, instead, added text in the discussion, which advocates that workers examine how the cumulative frequency plots change as a function of monitoring period. We also note how workers should be cognisant of long-term changes in animal behaviour that might occur e.g. seasonally (such as rutting by ungulates), which would affect the cumulative plots. If the referee/editor considers that this point should, nonetheless, be amplified, we are happy to do this.

Discussion

Line 395: A word appears to be missing here “deploy systems that about the 3-5% mass rule”.

Sorry. Corrected.

Line 404: Replace “animal” with “animal’s”.

Corrected.

Figures

Figure 4: The grey points and grey regression line are difficult to discern. I recommend replacing these with black or another darker color.

Done.

Supplementary Information
Line 160: Replace “coral” with “corral”.

Replaced.
